# The evolutionary life cycle of the polysaccharide biosynthetic gene cluster based on the *Sphingomonadaceae*

**DOI:** 10.1038/srep46484

**Published:** 2017-04-21

**Authors:** Mengmeng Wu, Haidong Huang, Guoqiang Li, Yi Ren, Zhong Shi, Xiaoyan Li, Xiaohui Dai, Ge Gao, Mengnan Ren, Ting Ma

**Affiliations:** 1 Key Laboratory of Molecular Microbiology and Technology, Ministry of Education, College of Life Sciences, Nankai University, Tianjin, China; 2 College of Agronomy & Resources and Environment, Tianjin Agricultural University, Tianjin, China; 3 Shanghai Majorbio Bio-pharm Biotechnology Limited Company, Shanghai, China

## Abstract

Although clustering of genes from the same metabolic pathway is a widespread phenomenon, the evolution of the polysaccharide biosynthetic gene cluster remains poorly understood. To determine the evolution of this pathway, we identified a scattered production pathway of the polysaccharide sanxan by *Sphingomonas sanxanigenens* NX02, and compared the distribution of genes between sphingan-producing and other *Sphingomonadaceae* strains. This allowed us to determine how the scattered sanxan pathway developed, and how the polysaccharide gene cluster evolved. Our findings suggested that the evolution of microbial polysaccharide biosynthesis gene clusters is a lengthy cyclic process comprising cluster 1 → scatter → cluster 2. The sanxan biosynthetic pathway proved the existence of a dispersive process. We also report the complete genome sequence of NX02, in which we identified many unstable genetic elements and powerful secretion systems. Furthermore, nine enzymes for the formation of activated precursors, four glycosyltransferases, four acyltransferases, and four polymerization and export proteins were identified. These genes were scattered in the NX02 genome, and the positive regulator SpnA of sphingans synthesis could not regulate sanxan production. Finally, we concluded that the evolution of the sanxan pathway was independent. NX02 evolved naturally as a polysaccharide producing strain over a long-time evolution involving gene acquisitions and adaptive mutations.

A gene cluster is a set of functionally related genes located in close physical proximity in a genome, and an operon, a more structured instance of a cluster, refers to a set of genes under common regulatory control^1^,^2^. Clustering of genes involved in the same metabolic pathway is a widespread phenomenon^3^,^4^. The genes of certain biosynthetic pathways for microbial polysaccharides^5^,^6^, such as cellulose^7^, alginate^8^, succinoglycan^9^, sphingans^10^,^11^ and xanthan^12^, form clusters and operons in the genome. Some models suggest a tendency for genes to cluster. For instance, the selfish operons model postulates that genes organized into a cluster can propagate by vertical transmission and horizontal transfer^13^,^14^,^15^, which might represent an instinct for self-preservation. The co-regulation model states that the formation of an operon promotes the production of gene products in equal measures^16^. The alternative explanation for cluster formation is known as the protein immobility model, which suggests that clustered genes produce local clusters of enzymes, and the physical proximity of the enzymes minimize the steady state level of reaction step intermediates and thus conserves energy and material required for growth and maintenance^17^. Thus, in the biosynthesis of a polysaccharide, genes might from clusters and operons. However, the genome structures within operons are unstable and shuffling of a genome structure is virtually neutral over long-term evolution^18^. The changes to operon structures can represent identical structure, similar structures (translocation, deletion, and insertion), destructed structures and complete loss^18^. In addition, Morgan *et al*.^1^ determined that the evolutionary of life cycle of operons occurs via the mechanisms of operon formation and death by gene insertion, deletion or rearrangement. To date, there has been no report of the evolutionary history of genes or clusters related to polysaccharide biosynthesis. Evolutionary research on these pathways is very difficult because of the clustering of responsible polysaccharide biosynthesis genes and exopolysaccharide (EPS) mutated strains with disrupted clusters during long-term evolution have received little research attention. Furthermore, the absence of recognized scattered polysaccharide synthetic pathway also makes exploring its evolution difficult.

The genus *Sphingomonas* belongs to the family *Sphingomonadaceae*^19^. Subsequently, the genus description was amended^19^,^20^,^21^,^22^. Based on phylogenetic analysis, polyamine patterns and fatty acid profiling, the genus *Sphingomonas* was subdivided into four genera: *Sphingomonas sensu stricto* and three new genera, *Sphingobium, Novosphingobium* and *Sphingopyxis*. The *Sphingomonadaceae* have great potential for biotechnological applications in bioremediation, the degradation of refractory contaminants and the production of valuable biopolymers. The biopolymers synthesized by strains of the *Sphingomonadaceae* have similar but not identical structures and are termed sphingans, including gellan, welan, rhamsan, S-88, S-7, S-198 and S-657^10^,^11^. Sphingans possess a similar linear repeating tetrasaccharide of [ → 4) α-L-Rha (1 → 3) β-D-Glc (1 → 4) β-D-GlcA (1 → 4) β-D-Glc (→1] (Supplementary Fig. S1)^10^. Sphingans are used in food, pharmaceutical, and other industries as a stabilizing, thickening, emulsifying, and gelling agents because of their excellent rheological characteristics^10^,^11^.

The general scheme of sphingan biosynthesis follows the Wzx/Wzy-dependent pathway as described for *Escherichia coli* group I and IV polysaccharide^23^,^24^. Biosynthetic pathways for sphingan S-88^25^, S-7^26^, gellan^10^,^27^, diutan^28^ and welan^11^,^29^ have been identified. Genes related to the assembly, polymerization and export of tetrasaccharide repeat units are clustered with same arrangement and orientation, and named the *spn* cluster^11^. Biochemical analysis indicated that the function of glycosyltransferase (GT) *spnB*^26^ and *spnK*^30^. Protein *spnC* and *spnE* potentially involved in secretion and chain length determination have also been analyzed biochemically^31^. The detailed enzymological functions of GT SpnL/Q, polymerase SpnG, and flippase SpnS remain unclear. In the sphingan gene cluster, the function of *spnI/J/F/M/N* are unknown, but might be related indirectly to sphingan biosynthesis^10^,^32^. The putative lyase SpnR might excise the mature polysaccharide chain from the outer membrane. Regulatory protein SpnA with regions homologous to sensor kinases and response regulator proteins^11^ is located far away from the sphingan cluster, thus its regulatory mechanism requires further research.

Newly isolated *Sphingomonas sanxanigenens* NX02 (DSM 19645), a Gram-negative and rod-shaped bacterium^33^, synthesizes a novel extracellular polymer sanxan, comprising a tetrasaccharide repeat unit of [→4) β-D-Man (1 → 4) β-D-GlcA (1 → 3) α-L-Rha (1 → 3) β-D-Glc (→1]^34^, which was very different from other sphingans (Supplementary Fig. S1). Sanxan has excellent thickening, shear thinning, gelling, and emulsification properties^35^, and has been used in China for years as drilling mud and as a thickening agent to recover petroleum by water flooding. In this study, we proposed a hypothetical evolutionary model for the distribution of genes for microbial polysaccharide biosynthesis, based on *Sphingomonas sanxanigenens* NX02 and the *Sphingomonadaceae*, comprising a lengthy cyclic process: cluster 1 → scatter → cluster 2. The genes responsible for sanxan biosynthesis prove the existence of a dispersive process. This evolutionary process was unstable and dynamic, and cluster 1 and 2 contained non-identical genes and rearrangement. In addition, we report the complete genome sequence of NX02 and the identification of a scattered pathway for sanxan biosynthesis, which partially explains sanxan’s specific structure. This is the first report that a scattered pathway could synthesize a polysaccharide. The analysis of the genome provided insights into its large genome size.

## Results and Discussion

### Genome features of *S. sanxanigenens* NX02

The complete genome of *Sphingomonas sanxanigenens* NX02 comprises a circular 6,205,897-bp chromosome and a 374,401-bp plasmid, with G + C contents of 66.8% and 64.9%, respectively. The genome of strain NX02 is the largest in the *Sphingomonadaceae* submitted to GenBank. The chromosome is predicted to contain 5,619 protein coding genes (CDSs), with an average size of 1,011 bp, nine rRNA operons and 58 tRNA genes. The plasmid contains 356 predicted CDSs with an average size of 903 bp, giving a coding intensity of 87.9%.

**Figure 1:**
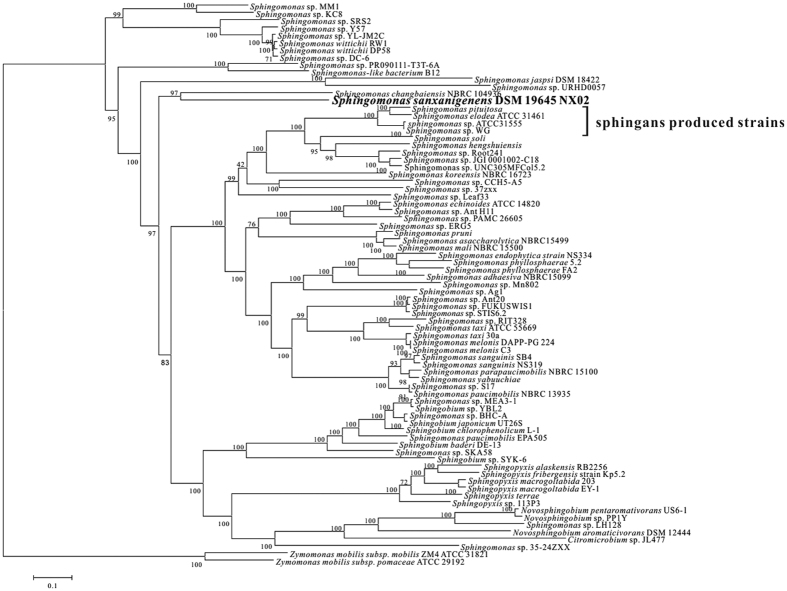
Neighbour-Joining phylogenetic tree of 77 *Sphingomonad* genomes constructed from concatenated nucleotide sequences of universally conserved genes using the Mega 6 tool. The numbers for the interior branches are bootstrap percentages. The scale bar indicates the number of substitutions per site. The four sphingan-producing strains are *S. pituitosa, S. elodea* ATCC 31461, *Sphingomonas* sp. ATCC31, and *Sphingomonas* sp. WG.

The phylogenetic relationships of *S. sanxanigenens* NX02 with 77 sequenced strains of the *Sphingomonadaceae* (submitted before April 2016) based on all core genes were analysed (Fig. 1). Most of these 77 sequenced strains, such as *Sphingomonas wittichii* RW1^36^, *Sphingobium chlorophenolicum* L-1^37^ and *Sphingobium japonicum* UT26S^38^, were reported to degrade persistent complex compound^39^. Besides NX02, only four sequenced strains were reported to produce polysaccharide like sphingans: *Sphingomonas elodea* ATCC 31461 (Gellan)^40^, *Sphingomonas* sp. ATCC31555 (Welan)^41^, *Sphingomonas* sp. WG (Welan)^42^ and *Sphingomonas pituitosa* (PS-EDIV)^43^. In addition, there was no EPS formation reported for the 72 non-sphingan producing strains. NX02 formed a separate evolutionary clade with *Sphingomonas changbaienesis* (isolated from Changbai mountains, China^44^), and the differentiation between them happened at an early stage of evolution. Despite being biopolymer-producing strain, NX02 was evolutionarily distant from the sphingan-producing strains.

### Genomic islands and horizontal gene transfer (HGT)

**Figure 2:**
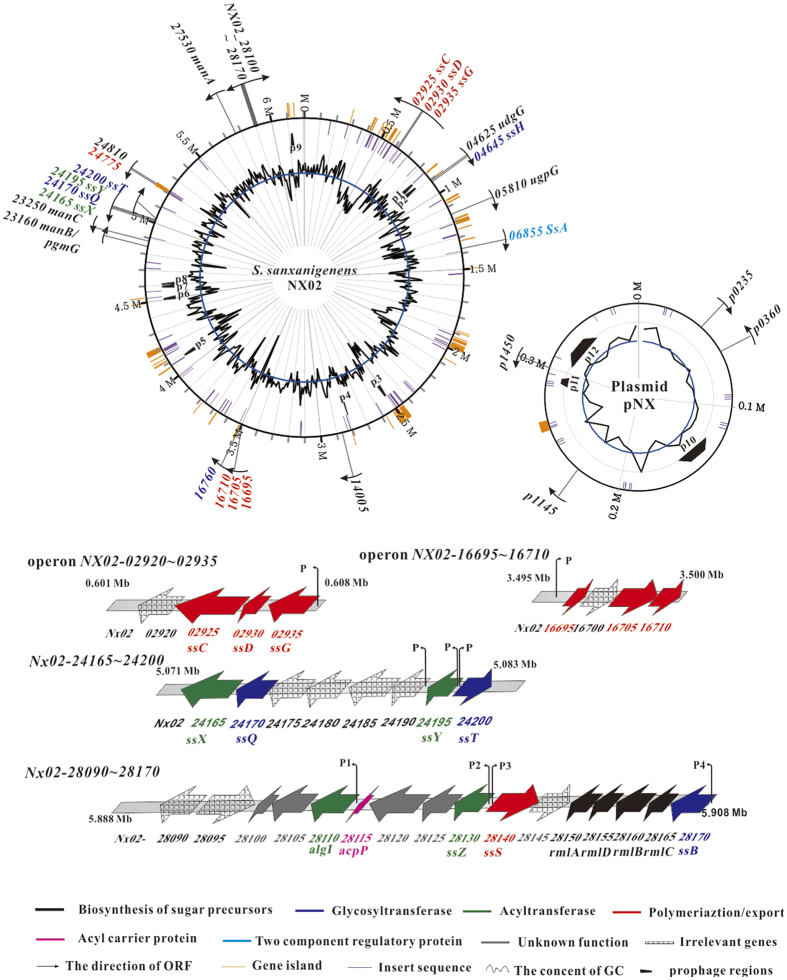
Genome features of *Sphingomonas sanxanigenens* NX02. The genome plot showing (from the outside to the centre) genes related to sanxan biosynthesis (different colours or symbols stand for genes with different functions), gene islands, genome size, insert sequence, prophage, and the GC content. The features of the four clusters related to sanxan synthesis are also shown.

The NX02 genome has undergone a number of HGT events assisted by phages and transposons. In all, 327 insertion sequences (ISs, E value < 1.00^e-20^), representing 93.8 kb (Supplementary Table S1), and 104 transposases were annotated in the NX02 genome. The most frequently identified were ISMdi7 (28 copies; 706 bp, 482 bp, 139 bp, and 212 bp appeared seven times, respectively) which originated from *Methylobacterium dichlormethanicum*. Twelve phages (215.6 kb) were found in the genome, nine of which were on the chromosome and three on the plasmid (Supplementary Table S2). An intact prophage was found in the chromosome (4076698–4105468), comprising 28.7 kb, with 35 CDS. In NX02, 45 gene islands (GIs), comprising 298 kb and 308 CDS, were identified, including transposases, transcriptional regulators and membrane proteins (Supplementary Table S3, Supplementary discussion). The distributions of ISs, prophages and GIs in the genome are shown in Fig. 2. Thus, NX02’s genome is very unstable and active, and the many ISs, transposases, GIs, and prophages would result in gene duplication, rearrangement, and loss, which accelerate genome evolution.

### Secretion systems and DNA uptake

Five typical secretion systems (SS) were found in the NX02 genome, including one T1SS, one T2SS, one T3SS, four T4SS, and one T6SS (Supplementary Fig. S2, Supplementary discussion). By comparison, *Sphingomonas* sp. ATCC 31555 contains one set of T1SS–T4SS, and *S. elodea* ATCC31461 and *S. wittichii* RW1 only have one T1SS, T2SS and T4SS. The T1SS of NX02 comprised a single copy protein TolC (outer membrane protein, OMP), 11 copies of HlyD as the MFP (membrane fusion protein) and two copies of ABC (ATP-binding cassette, which showed low homology with the HlyB protein, but was near the MFP) and might be responsible for drug resistance. The T3SS comprised 11 gene products from *NX02_15795* to *NX02_15875*, which all shared higher homology and were classified as T3SS components form *Sphingomonas* sp. SKA58. This cluster was considered a “pathogenicity island”. Four sets of T4SSs are present in NX02, namely T4SS-1 (*NX02_p0495* to *NX02_p0580*), T4SS-2 (*NX02_09735* to *NX02_09825*), T4SS-3 (*NX02_11790* to *NX02_11845*), and T4SS-4 (*NX02_19725* to *NX02_19775*). T4SS-1 is on the plasmid, while T4SS-1, -2 and -3 are on the chromosome. The T6SS of NX02 comprises 15 genes in the *imp* operon, including *vgrG, hcp, vasU*, and *clpV*. Interestingly, 12 of the NX02 T6SS genes are most similar to *imp* genes from *Sphingomonas sp*. S17.

Naturally competent bacteria use certain proteins to take up DNA. Parts of this common competence system share homology with proteins that are involved in the assembly of type IV pili and type II secretion systems, and form a structure that spans the cell envelope partially^45^,^46^. Interestingly, NX02 has a T2SS and four sets of T4SS, suggesting that NX02 has greater capacity to take up exogenous DNA.

Thus, the secretion systems of NX02 probably play a critical role in HGT, permitting adaption to the environment, and driving bacterial. These multiple TSSs might explain the huge genome. In addition, the ISs, transposases, GIs, and prophages could also enlarge the genome by multiple gene duplication.

### The identification of the biosynthetic pathway of the biopolymer sanxan

The biosynthetic pathways of sphingans are similar to those of group 1 and 4 capsule polysaccharides from *E. coli*^23^,^24^. The sanxan biosynthetic pathway also comprises a multi-step Wzx/Wzy-dependent process^11^,^23^,^24^, which is divided into three sequential steps: (a) sugar-activated precursors are synthesized simultaneously; (b) tetrasaccharide repeat units are assembled into the inner membrane; and (c) the repeat units are polymerized and exported through the outer membrane. The detailed process and enzymes involved in sanxan synthesis are as follows.

#### Genes and enzymes involved in the formation of nucleotide sugar precursors

**Figure 3:**
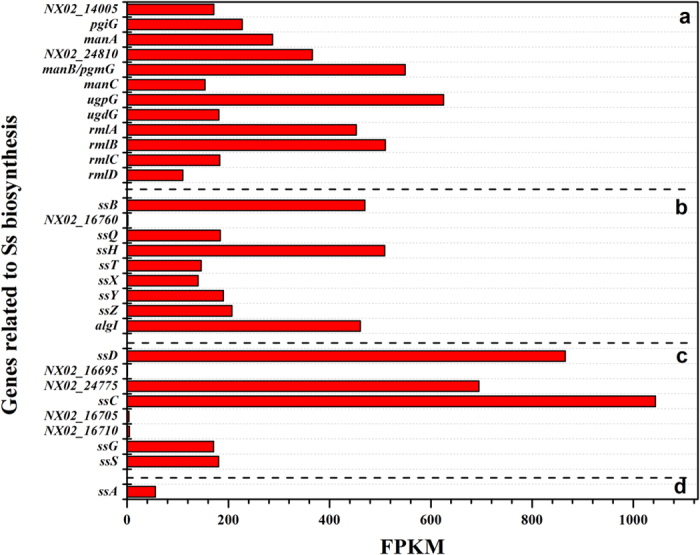
FPKM (Fragments Per Kilobase of exon per Million mapped reads) values of genes related to sanxan biosynthesis on NK medium. “a” stands for genes related to precursor biosynthesis; “b” stands for genes responsible for the assembly of the repeat units; “c” indicates genes related to the polymerization and export of sanxan polysaccharides; “d” is the *ssA* gene.

The tetrasaccharide repeat units of sanxan are synthesized from activated UDP-D-glucose, UDP-D-glucuronic acid, GDP-D-mannose, and dTDP-L-Rhamnose (Supplementary discussion). The *pgmG* gene, encoding a phosphoglucomutase (EC 5.4.2.2), is represented by *NX02_14005* and *NX02_23160*^47^. Their FPKM (Fragments Per Kilobase of exon per Million mapped reads) values were both high (Fig. 3). The *NX02_14005* disruptant had an Ss^+^ phenotype like the wild-type strain, while the *NX02_23160* mutant showed an Ss^−^ phenotype in NKS medium (figure not shown). The sanxan yield of the *NX02_23160*-deficient strain in fermentation broth observably decreased, and its overexpression resulted in a 17 ± 0.3% increase sanxan production^47^. Therefore, *NX02_23160* encodes the main phosphoglucomutase, PgmG, while NX02_*14005* might be an alternate protein.

The *ugpG* gene, encoding a UDP-glucose pyrophosphorylase (EC 2.7.7.9), which catalyses the reversible conversion of glucose-1-phosphate and UTP into precursor UDP-D-glucose and diphosphate^48^, is *NX02_05810*. The *ugdG* gene, encoding a UDP-glucose dehydrogenase (EC 1.1.1.22), which converts UDP-glucose into the activated precursor UDP-D-glucuronic acid, is *NX02_04625*^49^. The enzymes required for the synthesis of precursor dTDP-L-Rhamnose are TDP-glucose pyrophosphorylase (RmlA, EC 2.7.7.24, NX02_28150), dTDP-glucose 4,6-dehydratase (RmlB, EC 4.2.1.46, NX02_28160), dTDP-4-dehydrorhamnose 3,5-epimerase (RmlC, EC 5.1.3.13, NX02_28165), and dTDP-4-dehydrorhamnose reductase (RmlD, EC 1.1.1.133, NX02_28155). The wild-type strain deleted for the *rml* cluster was not obtained despite screening a large number of mutants. However, the mutant strain could be obtained in a glucosyl-isoprenylphosphate transferase-deficient strain.

The activated precursor GDP-D-mannose is synthesized by mannose-6-phosphate isomerase (ManA, EC 5.3.1.8, NX02_27530), phosphomannomutase (ManB, EC 5.4.2.8, bifunctional gene NX02_28160), and Mannose-1-phosphate guanylyltransferase (ManC, EC 2.7.7.22, NX02_23250). These related genes were scattered among the genome. Identity or similarity analysis between proteins related to precursor synthesis from *S. sanxanigenens* and *S. elodea* ATCC 31461, *Sphingomonas* sp. ATCC 31555, and *Sphingomonas* sp. ATCC 53159 are shown in Table 1.

#### Genes and enzymes involved in the assembly of the tetrasaccharide repeat unit

The synthesis of activated precursors was followed by the formation of the tetrasaccharide repeat unit by sequential transfer of the sugar and acyl donors to an activated lipid carrier by glycosyltransferases (GTs) and acyltransferases (ATs), according to the structure of sanxan (Lipid-P-P ← β-D-Glc ← α-L-Rha ← β-D-GlcA ← β-D-Man, Supplementary Fig. S1). The tetrasaccharide repeat units were assembled on a lipid carrier comprising the C_55_-isoprenylphosphate carrier (IP), which was similar to the group I capsular polysaccharide in *E. coli*^23^,^24^.

**Figure 4:**
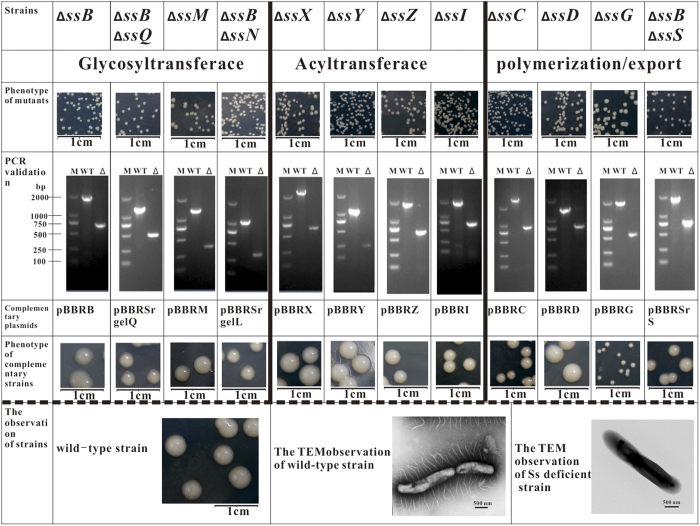
The phenotypes and genotypes of different mutants and complementary strains, together with transmission electron microscopy observations of wild-type and sanxan-deficient strains.

The priming glycosyltransferase (SsB) annotated as glucose-1-isoprenylphosphate transferase, which transfers glucose-1-phosphate from UDP-glucose to the lipid carrier IP, was encoded by *NX02_28170*. The *ssB* gene is located upstream of the *rml* cluster and shares a common promoter (Fig. 2). The s*sB*-deficient strain showed an Ss^−^ phenotype and was complemented by plasmid pBBRssB or pBBRgelB. The genotype and phenotype of the mutant and complementing strains are shown in Fig. 4. Protein SsB has four predicted N-terminal transmembrane regions and one at Leu_280_ to Val_301_; its C-terminus is predicted to be cytoplasmic. SsB is homologous to GelB from *S. elodea* ATCC31461 (41.6%)^28^, WelB from *Sphingomonas*. sp. ATCC 31555 (43.4%), and SpsB from *Sphingomonas*. sp. ATCC 53159 (44.1%), Table 1. In addition, another gene, *NX02_16760*, was also predicted as glucose-1-isoprenylphosphate transferase, however its FPKM value was low, and the mutant strain had the Ss^+^ phenotype with unchanged sanxan production (Supplementary Fig. S3). Thus, under most circumstances, *NX02_16760* is probably irrelevant to sanxan biosynthesis.

In the biosynthesis of gellan, welan, diutan, or S-88, genes related to assembly, polymerization, and export are clustered with almost the same arrangement^11^. However, similar gene clusters were not found within 50 kb of *ssB*. Other GTs were located in separate loci in the genome. According to the CAZY database and gene annotation, 33 GTs were found in the chromosome. The purified yields of strains deficient for these GTs and their FPKM values are shown in the Supplementary discussion and Supplementary Fig. S3. Among of all 33 genes, *NX02_24170, NX02_24200* and *NX02_04645* were special (Supplementary discussion).

Deletion of *NX02_24170* and *NX02_24200* failed in the wild-type strain, while a mutant could be obtained in strain NX02 (∆*ssB*). Complementation experiments showed that strain NX02 (∆*ssB*, ∆*24170*) had an Ss^+^ phenotype when plasmid pBBRSrgelQ was transferred (Fig. 4). *NX02_24170* was homologous to *gelQ* (only 22.6% identity, Table 1) and it was named as *ssQ*. SsQ is demonstrated to be the second glycosyltransferase that transfers rhamnose from dTDP-L-rhamnose to IPP ← glucose. Strain NX02 (∆*ssB*, ∆*24200*) could be complemented by plasmid pBBRSrgelL. GelL catalyses the addition of β-D-glucose to β-D-glucuronic acid in gellan synthesis^32^. However, this connection type (β-D-GlcA ← β-D-Glc) does not exist in the structure of sanxan (Supplementary Fig. S1). The C2-epimer of β-D-glucose is β-D-mannose, thus *NX02_24200* might be *ssT*, whose product adds β-D-mannose to β-D-glucuronic acid to form a new type of intermediate (β-D-GlcA ← β-D-Man). Mutation of *ssT* and *ssQ* was lethal if sanxan synthesis had been initiated on the lipid carrier, which was similar to the knockout of gene *gumB/C/E/M/J* in *Xanthomonas campestris*^12^.

Sanxan production was blocked significantly by inactivation of *NX02_04645*. The phenotype of NX02 (∆*04645*) was Ss^−^ and could only be recovered by complementation with plasmid pBBR04645, but not by any other glycosyltransferase in the *gel, wel* or *sps* clusters. It is speculated that a glycosyltransferase, SsH, encoded by *NX02_04645*, catalyzses the connection of β-D-glucuronic acid to α-L-Rhamnose, a connection that does not exist in other sphingans. The gene loci of these four GTs are shown in Fig. 2. Therefore, according to the repeat unit of sanxan, four monosaccharides are transferred to the lipid carrier by SsB, SsQ, SsH, and SsT in that order.

**Figure 5:**
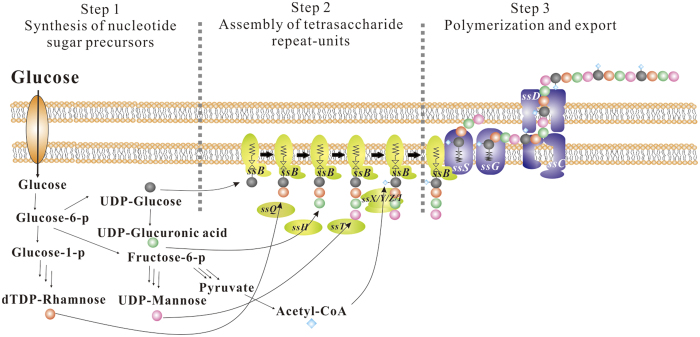
Schematic diagram of sanxan biosynthesis, modified according to Fialho *et al*.^10^. The first step is the synthesis of nucleotide sugar precursors from glucose. The second step is the assembly of the tetrasaccharide repeat unit by the sequential activity of SsB, SsQ, SsH, and SsT glycosyltransferases, and SsX, SsY, SsZ, and SsI acyltransferases. The third step, comprising polymerization and export of the final product, is accomplished by SsS, SsG, SsC, and SsD.

Four ATs genes were found near the GTs and other related genes: *NX02_24165, NX02_24195, NX02_28130*, and *NX02_28110*. The phenotypes of the mutants of these genes were all Ss^−^ and they were complemented by their respective plasmids (Fig. 4). These four ATs were all membrane proteins possessing at least 10 transmembrane domains. These enzymes add acyl groups to the integrated tetrasaccharide repeat unit to prepare for subsequent polymerization and export process. However, the detailed mechanism for the addition of the acyl to the repeat unit is unclear. The schematic diagram of the process of sanxan biosynthesis is shown in Fig. 5.

#### Genes and enzymes involved in the polymerization and export of repeat units

The polymerization and export of sanxan repeat units is a Wzx/Wzy-dependent process. No flippase (termed SsS in *S. sanxanigenens*) was found in the genome (because of lower identity) by the programme tblastn in Bioedit software based on the *gel, wel*, and *dps* clusters. While the protein encoded by *NX02_28140* (K03328, FPKM 180.78) was a polysaccharide transporter, it showed highest (40%) identity with RfbX, which is involved in the export of the O-antigen and lower identity with GelS (14.7%), WelS (18.7%) and DpsS (16.7%), respectively (Table 1). The mutated strain could only be obtained in an *ssB*-deficient strain, while it was lethal in the wild-type strain. The phenotype of strain (∆*ssB*, ∆*28140*) could be complemented by recombinant plasmid pBBRSr28140 (Fig. 4). Thus, the protein encoded by *NX02_28140* is SsS.

Two operons encoding polysaccharide co-polymerases, a tyrosine phosphatase, and an outer membrane auxiliary protein were found in the genome, they were *NX02_02920*–*02935* and *NX02_16695*–*16710* (Fig. 2). *NX02_16695/02930, NX02_16705/02925*, and *NX02_16710/02925* were similar to *wza, wzc*, and *wzb*, respectively. Notably, *NX02_02925* and *NX02_16705/16710* were different. *NX02_16705/16710* are similar to *gelC/gelE* in *S. elodea* ATCC31461, which exhibited a common genetic organization in the *Sphingomonas* genus and is homologous to *NX02_02925*, the product of which comprises only one polypeptide instead of two independent polypeptides like GelC/GelE^31^. The comparison of their FPKM (Fig. 3) showed higher values for operon *NX02_02920*~*02935*. The other operon might be silent or suppressed by certain factors, which was confirmed when its inactivation did not affect the sanxan yield, the viscosity of the fermentation broth, and the product composition. In addition, *NX02_24775*, located in a gene island, was also analogous to polysaccharide export protein Wza. Its mutant strain had an Ss^+^ phenotype. The markerless deletion of operon *NX02_02920*~*02935*, or of each gene, affected the phenotype (Ss^−^) significantly, reducing the yield of sanxan (Fig. 4). The polypeptide named SsC, encoded by *NX02_02925*, was identified as the autophosphorylating tyrosine kinase involved in polysaccharide chain length determination^31^. The Ss^−^ phenotype of strain NX02 (∆*ssC*) could not be completely recovered by the transformation with plasmid pBBRssC and the yield of sanxan was slightly decreased. SsC comprises 751 amino acids, of which M_1_ to R_489_ from the N-terminus are homologous to GelC (15.6%), WelC (17.6%), and DpsC (16.6%), and E_490_ to G_751_ from the C-terminus are similar to GelE (20.1%), WelE (22.8%), and DpsE (22.1%) (Table 1); these two segments are homologous to the activator domain and the kinase domain of SsC, respectively. SsC was predicted to have two transmembrane α-helices, TM1 and TM2, located at W_54_ to T_76_ and V_469_ to A_488_. *NX02_02930*, named as *ssD*, is homologous to *spnD*, the product of which is an OMA protein homologue that is responsible for the export of sanxan chains. NX02 (∆*ssD*, Ss^−^) could be complemented by plasmid pBBRssD to an Ss^+^ phenotype. SsD was not predicted to have a transmembrane helix, like GumB; however, GelD, WelD, and DpsD all have one helix in their N-terminus. Although three copies of *wza* and two copies of *wzc* homologous genes were found in the genome, only one gene was responsible for sanxan biosynthesis.

The polymerase related to sanxan biosynthesis was named SsG. *NX02_02935*, annotated to encode an O-antigen polymerase, was identified by browsing the whole genome. Its deletion reduced the production of sanxan drastically, thus *NX02_02935* was gene *ssG*. However, multicopy expression of *ssG* in NX02 (∆*ssG*) did not recover the Ss^−^ phenotype after transformation with pBBRSsG (Fig. 4). This might be because a balanced expression level of *ssC* and *ssG* is necessary to assemble the membrane protein complex correctly. In addition, the Ss^−^ phenotype of strains NX02 (∆*ssC*), NX02 (∆*ssD*), and NX02 (∆*ssG*) could not be complemented by plasmids pBBRgelC/E (welC/E), pBBRgelD (welD), and pBBRgelG (welG), respectively. Thus, the polysaccharide biosynthesis process might show catalytic specificity for the polymerization and export of the repeat units.

#### Regulatory gene

A multi-sensor hybrid histidine kinase SsA, encoded by *NX02_06855*, is homologous with GelA (60.5% identity) from *S. elodea*. It contains 797 amino acids and two transmembrane helices in the N terminus: G_22_ to G_44_ and G_49_ to F_66_. Our knockout experiments showed that mutation of *ssA* did not affect the yield of sanxan. In addition, the expression levels of genes related to sanxan synthesis between NX02 (∆*ssA*) and the wild-type were similar or only slightly altered (Supplementary Fig. S4). Thus, the deletion of *ssA* did not affect the expression levels of related genes. Therefore, the positive regulator of gellan, welan and other sphingans synthesis could not regulate sanxan production.

#### Sanxan is a capsular polysaccharide

The sphingans, such as gellan and welan, are structurally related EPSs secreted by a group of the genus *Sphingomonas*^9^. A lyase, SpnR, found in the *spn* cluster, released the polysaccharide from the outer membrane into extracellular environment. In addition, the deletion of *dpsM* and/or *dpsN* leaded to more easy removal of the polysaccharide from the cells^50^. However, *spnR, dpsM*, and *dpsN* were all not found in NX02 genome. The surfaces of bacteria cells of *S. elodea* ATCC31461 and *Sphingomonas*. sp. ATCC 31555 were both smooth in YEME medium, while in NX02, sanxan was spread over the cell surface (Supplementary Fig. S5a,b,c). When plasmid pBBRgelR was overexpressed in NX02, sanxan was released from the NX02 cell surface and the capsular-free cells tended to gather together (Supplementary Fig. S5d). These results suggested that sanxan is a capsular polysaccharide and that such a lyase does not exist in NX02 genome.

#### Comparisons of the biosynthetic pathways of sanxan, sphingans, and xanthan

All gene loci related to sanxan biosynthesis are shown in Supplementary Fig. S6, and are scattered over the whole genome. By contrast, the genes responsible for the assembly, polymerization, and export in all sphingans were clustered, with a uniform arrangement^6^,^11^, although they are transcribed by several promoters. Twelve *gum* genes form an operon under the control of a single promoter. Four *rml* genes were clustered and arranged as the sequence *rmlC*-*B*-*D*-*A*, showed the same order with *S. wittichii*, while those in sphingan-producing strain were all *rmlA*-*C*-*B*-*D*^11^. In addition, the *spnI, spnJ, spnF, spnM, spnN*, and *spnR* genes were not found in the NX02 genome, which suggest these six genes were not essential for the biosynthesis of sanxan during the long-term evolution. Two submits of protein SsC are homologous with SpnC and SpnE. Four ATs were also found in NX02 genome. In brief, the biosynthetic pathway of sanxan was more like a “patchwork” of dispered gene elements from different locations. Thus, because of the obviously different pathways and the low similarity of the related genes, the structure of sanxan is distinct.

### The evolutionary analysis of the arrangement of genes related to sanxan biosynthesis

**Figure 6:**
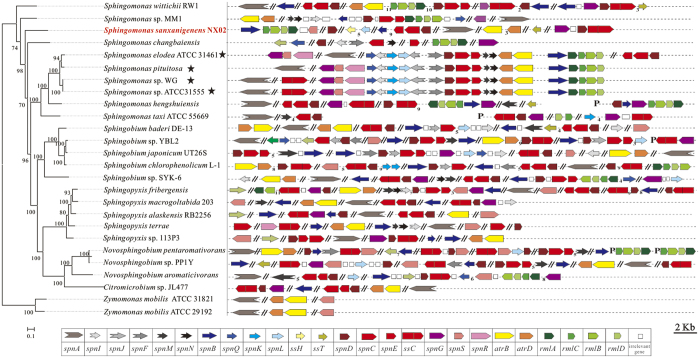
Genes related to assembly, polymerization and export, and their distribution in 26 genomes. The phylogenetic relationships of *S. sanxanigenens* NX02 with 25 sequenced strains of the family *Sphingomonadaceae* (including 21 other sequenced genomes and four sphingan-producing genomes) were constructed using the Neighbor-Joining method based on all core genes. Related genes were predicted by gene annotation and homology alignment against *ss* and *gel* genes. “★” indicates the sphingan-producing strains.

Compared with sphingans, the capsular polymer sanxan possesses a specific structure, specific properties^34^, and scattered biosynthetic genes. In addition, NX02 is phylogenetically distant from sphingan producing strains, and the homology between the *ss* genes (all genes related to sanxan biosynthesis) and *spn* genes was very low. Furthermore, many unstable genetic elements exist in the NX02 genome. To obtain clues to the evolutionary process of the arrangement of genes related to sanxan biosynthesis, we analysed all those genes in *Sphingomonadaceae* strains with completely sequenced genomes, based on gene annotation and homology alignment against *ss* and *gel* genes. The genes related to the assembly, polymerization and export and its distribution in 26 genomes, include 22 completed sequenced genomes and four genomes of sphingan producing strains (*Sphingomonas* sp. ATCC 31555, *Sphingomonas* sp. WG, *S. elodea* ATCC 31461 and *Sphingomonas pituitosa*) are shown in Fig. 6. The arrangement of genes in the four sphingan-producing strains was approximately consistent, except in one case where the location of *gelG/S/R* was distant from the main area. Although 21 strains were reported as non-sphingan producing strains, most genes existed in a dispersed form in their genomes, which was similar to NX02. For example, *S. wittichii* RW1 possessed most genes except for one GT; and *Sphingomonas* sp. MM1, *S. japonicum* UT26S, *Sphingobium* sp. SYK-6, and *N. aromativivorans* retained many traces of the *spn* cluster. Genes *spnD/C/E* were always clustered and were present as multicopies, which suggests their evolutionary diversity. The order of *rml* genes in NX02 was same as that in sphingan-free strains.

Therefore, we deduced that in the progenitor of NX02, *ss* genes were scattered over the genome and were incomplete like other non-sphingan producing strains, and the phenotype of progenitor NX02 might have been Ss^−^. Sanxan could be produced when some adaptive mutations and HGTs happened during long term evolution. The large number of GIs, ISs, transposases, and prophages would facilitate this process of evolution. The *ssH* gene is located between a GI and an IS (Fig. 2), thus it might be an exogenous gene obtained by HGT. Judging by their specificity, adaptive mutations might have occurred in the nucleotide sequence of the *ssC/D/G/S* genes. The evolution of the synthetic pathway for sanxan was independent and very different from that of sphingan. Consequently, the structure of sanxan is obviously different from the sphingans, and the common positive regulator SsA of sphingans could not regulate sanxan production. Based on the above, we concluded that *S. sanxanigenens* NX02 was a natural polysaccharide producing strain that evolved over a long-time.

### A hypothetical evolutionary model related to the polysaccharide biosynthetic cluster

**Figure 7:**
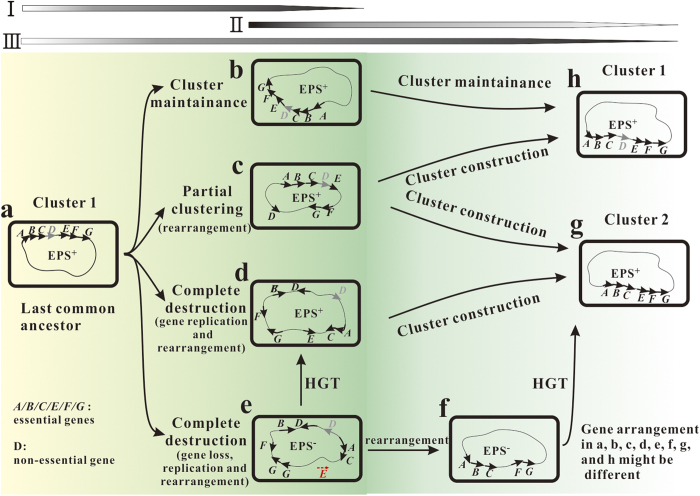
Hypothetical scheme for the lengthy cyclic evolutionary process of the biosynthetic pathway for microbial polysaccharides. There are many possible forms (**a**–**g**) of the evolutionary process (I–III). “A~G” stands for different genes related to polysaccharide biosynthesis.

Genes responsible for polysaccharide biosynthesis were always clustered^5^,^6^,^7^,^8^,^11^. Research into the evolution of their pathways is difficult because the phenotype of a strain would change to EPS^−^ when the cluster was destroyed during the long period evolution, and these mutants with the EPS^−^ phenotype would not arouse researchers’ attention. Itoh *et al*.^18^ pointed out that the shuffling of a genome structure was virtually neutral over long-term evolution and that gene order in operons was unstable. This evolutionary process also has been demonstrated by analysis of the *cps* (capsular polysaccharide synthesis) gene clusters within *Klebsiella* spp., in which many shuffling phenomena such as lateral gene transfer, truncation, and transposition, were observed^5^. Therefore, genes in a cluster are also not constant; events such as translocation, deletion, and insertion happen frequently (Fig. 7I). Subsequently, cluster 1 changed to a b, c, and d forms or with a different gene arrangement, as described in Fig. 7, and polysaccharides could not be produced after complete destruction of the cluster (Fig. 7e). However, a metabolic pathway should be regulated for the effective use of energy, with only the related genes being organized into operons or clusters^17^, and this gene cluster could also promote the lateral transfer of the phenotype^1^,^13^,^14^ (Fig. 7II). In addition to these two models of cluster formation, other models have been proposed for the formation of operons, a more structured instance of cluster, for example the Natal Model^51^, Fisher Model^2^, and Co-regulation Model^16^. Furthermore, an evolutionary model for the origin and evolution of proteobacterial histidine biosynthetic operons described a piecewise building process from single genes to one operon^52^. After long-term evolution, a new cluster 2 would appear that did not include the non-essential gene *D* or essential genes with different arrangement (Fig. 7g). Therefore, the evolutionary process of the biosynthetic pathway for microbial polysaccharide might be proposed as a lengthy cyclic process: cluster 1 → scatter → cluster 2 (Fig. 7III). In this process, genes in cluster 1 and cluster 2 were always not identical. The biosynthetic pathway of sanxan proved the existence of the scatter process. This process would lead the appearance of many new species.

Based on *Sphingomonadaceae*, the gene cluster of the sphingan-producing strains might be the last common ancestor (Fig. 7). With increasing time, some genes in this cluster were translocated, deleted, and the cluster might have been broken or lost. Strong evidence for this hypothesis is provided by the existence of partial non-essential spn*I/J/F/M/N/R* genes in most sphingan-free strains, such as *Sphingomonas* sp. MM1 and *S. japonicum* UT26S, and the translocation of the *gelG/S/R* genes in the *gel* cluster (Supplementary Fig. S6). The phenotype of EPS^+^ was lost during this process, and many new strains appeared. NX02 might have undergone this evolutionary journey, and losing the six *spnI/J/F/M/N/R* genes in the process. Subsequently, to defend against extreme environments, NX02 acquired some genes by HGT or adaptive mutations to produce a capsular polysaccharide that is different from the sphingans. Thus, the organization of *ss* genes demonstrated that clustering is not essential for polysaccharide production. However, as a more efficient form, a new cluster or operon will appear after long-term evolution. The new cluster might not contain the *spnI/J/F/M/N/R* genes, and will be stable under the pressure of severe environments, or might be destroyed because of the unstable genome structure. The putative evolution process of NX02 could be described as: a → e → d → g (Fig. 7). It is likely that after long-term evolution, another polysaccharide-producing strain like NX02 in the *Sphingomonadaceae* will appear.

## Methods

### Strains, plasmids, media, and culture conditions

The bacterial strains and plasmids used in this work are listed in Table 2. *Escherichia coli* strains were grown in Luria Bertani (LB) medium. *Sphingomonas Sanxanigenens* NX02 was cultured at 30 °C on NKG (NK medium with 1.5% glucose; NK: 0.5% peptone, 0.3% beef powder, 0.1% yeast extract and 1.5% agar 15.0 g, pH 7.0), NKS (NK medium with 8% sucrose), and YEME medium (0.25% yeast extract, 0.025% malt extract) that was developed to reduce sphingan production and improve cell suspension^28^. Antibiotics were used at the following concentrations (μg/mL): tetracycline (Tc; 10), kanamycin (Km; 25), and Chloramphenicol (Cm; 25). For sanxan fermentation, cells were grown in medium consisting of: 4% glucose, 0.02% yeast extract, 0.12% K_2_HPO_4_, 0.2% NaNO_3_, 0.1% CaCO_3_, 0.0005% FeSO_4_, 0.04% NaCl, and 0.05% MgSO_4_ (pH 7.5)^55^. Peptone, beef powder, yeast extract, agar, and other chemicals were purchased from Dingguo Limited (Tianjin, China).

### Genome sequencing and analysis of ORFs from *S. sanxanigenens* NX02

The complete genome sequence of *S. sanxanigenens* NX02 has been deposited in GenBank under the accession nos. CP006644 and CP011450. Whole genome sequencing was performed using the Illumina Hiseq 2000 and Pacific RSII platforms. The genome was assembled using 1.2 GB Illumina paired-end reads, 1.7 GB Illumina mate-paired reads, and 53.8 MB PacBio reads. Sequence quality assessment and assembly were performed with a quality of <1 error in 100,000 bases using PHRAP and Consed. Error correction of the PacBio reads was performed using the Illumina reads. Genes were predicted using Glimmer3^56^ and tRNAscan-SE^57^, and annotated by searching against the nr protein database of GenBank using blastp, with E values less than 1.00^e-5^. A Neighbour-Joining phylogenetic tree was constructed in Mega6^58^. Genomic islands were predicted using IslandViewer, which integrated the IslandPath-DIMOB and SIGI-HMM algorithms^59^. The ISs were identified and classified using the ISfinder database^60^. Percent identities or similarities between amino acid sequences were calculated using the online programme EMBOSS Needle, (http://www.ebi.ac.uk/Tools/psa/emboss_needle/). The prediction of transmembrane helices in proteins was performed using the TMHMM Server v.2.0 (http://www.cbs.dtu.dk/services/TMHMM/). Genes associated with certain pathways were analysed using the Kyoto Encyclopedia of Genes and Genomes (KEGG) database (http://www.genome.jp/kegg/). The glycosyltransferases of *S. sanxanigenens* NX02 were analysed using the Carbohydrate-Active Enzymes database (CAZY; http://www.cazy.org/) and the NCBI database.

### Markerless gene knockout and complementation

Genes were inactivated by double-crossover homologous recombination. The upstream and downstream flanking sequences (approximate 1.5 kb) of genes were spliced using overlap extension PCR. The primers used to amplify the flanking fragments of the target genes contained SacI, XbaI, or PacI restriction sites; all primers are shown in Supplementary Table S4. PCR products of the respective genes were digested with the appropriate restriction enzymes, ligated into a suicide vector, pLO3^53^ (Table 2), and used to transform *E. coli* S17. The respective recombinant plasmids were transferred to *S. sanxanigenens* NX02 wild-type strain or NX02 (∆ *ssB*) strain using biparental filter mating at 30 °C for 12 h on NKG medium without antibiotics. The single crossover mutants were selected on NKG medium containing 10 μg/mL Tc and 25 μg/mL Cm. The knockout mutants were then isolated on NKS medium with 25 μg/mL Cm, followed by PCR screening using the verification primers. The large fragments were deleted using the same procedures.

To identify the function of a deleted gene, complementation tests were performed as follows. The targeted genes related to sanxan synthesis, or specific genes from *S. elodea* ATCC31461 or *Sphingomonas*. sp. ATCC 31555, were amplified and ligated into the broad host range expression vector pBBR1MCS-2^54^ or pBBRssB (Table 2), and the recombinant vectors were transferred into the respective mutant strains of *S. sanxanigenens* NX02 using biparental conjugation. Primers used to construct expression vectors are shown in Supplementary Table S4. The recombinant expression vectors were verified by PCR screening and DNA sequencing, and the recombinant strains harbouring the plasmids were selected using PCR. The genome of *S. sanxanigenens* NX02 was extracted using an AxyPreP™ Bacterial Genomic DNA Miniprep Kit (Axygen, Hangzhou, China). Plasmid DNA was purified from *E. coli* using the Axyprep™ Plasmid Miniprep Kit (Axygen). PCR products were purified using a DNA Gel Extraction Kit (Axygen) and a PCR Cleanup Kit (Axygen).

### RNA isolation and chain specific transcriptome sequencing

Large amounts of sanxan accumulated around the cells on NK medium. The crude total DNA-free RNA of *S. sanxanigenens* NX02 was extracted using the RNAiso Plus (Takara, Dalian, China) and RNAprep Pure Cell/Bacteria Kit (Tiangen, China) when the strain reached cultured at logarithmic phase in NK medium. Total RNA quality was assessed using a gel electrophoresis BioDrop Cuvette (BioDrop, United Kingdom). rRNA was depleted from the total RNA using a Ribo-Zero Magnetic kit (Epicentre, Madison, WI, USA). Chain specific transcriptome sequencing of the double-stranded cDNA was performed following the Illumina workflow on a Hiseq 2500 (Illumina) using the Truseq PE Cluster Kit v3-cBot-HS (Illumina) and the cBot instrument (Illumina)^61^. A total of 17,549,424 reads were generated that resulted in 285-fold sequencing coverage. The sequence quality satisfied the criterion of <3 error in 10,000 bases. The number of fragments per kilobase of exon per million mapped reads (FPKM) was calculated to measure expression levels of the genes by RSEM (RNA-Seq by Expectation-Maximization, http://deweylab.biostat.wisc.edu/rsem/)^62^,^63^. Operon identification was also performed using chain specific transcriptome sequencing^64^. If multiple genes share the same transcriptional start site and termination site after expanded sweep, these genes will belong to an operon^64^.

### cDNA synthesis, and qRT-PCR

Total RNA (1.5 μg) was reverse-transcribed using a Quantscript RT Kit (Tiangen, China), according to the manufacturer’s protocol. The relative expression analysis of genes related to sanxan biosynthesis in different strains was performed using the quantitative RT-PCR with a MyiQ™ two-colour real-time PCR detection system (BIO-RAD laboratories) with the Bestar^®^ SybrGreen qPCR mastermix (DBI, Bioscience Inc., Germany). The primers were designed using the OLIGO software and the length of amplicons was between 100 and 200 bp. The primer sequences used in qRT-PCR are listed in Supplementary Table S5. The endogenous reference gene was 16srRNA. Standard deviations were calculated from three PCR replicates and the relative abundance of the genes was determined using the comparative Ct method.

### Analysis of fermentation broth

The extraction of sanxan from the fermentation was performed according to a previously published method^35^. The viscosity of the sanxan solution was measured using a Brookfield viscometer DV_II + (USA) equipped with a no. 64 spindle at a shear rate of 60 rev/min.

### Electron microscopy

Strains with different phenotypes or genotypes were prepared for transmission electron microscopy (TEM; Hitachi, Tokyo, Japan). NX02, *Sphingomonas* elodea ATCC31461 and *Sphingomonas* sp. ATCC31555 strains were cultured in YEME medium at 30 °C for 18 h. Then strains were collected, washed with phosphate buffer twice to remove impurities, and 1 μl of cell suspension at an appropriate concentration was dropped onto Holey carbon Film and observed directly^65^.

## Additional Information

**How to cite this article:** Wu, M. *et al*. The evolutionary life cycle of the polysaccharide biosynthetic gene cluster based on the *Sphingomonadaceae. Sci. Rep.* **7**, 46484; doi: 10.1038/srep46484 (2017).

**Publisher's note:** Springer Nature remains neutral with regard to jurisdictional claims in published maps and institutional affiliations.

## Supplementary Material

Supplementary Information

## Figures and Tables

**Table 1:** Identity or similarity analysis between amino acids used for sanxan biosynthesis and the proteins responsible for gellan, welan, and diutan, respectively.

Sanxan	Amino acids	Predicted function	% Identity (% Similarity)			Accession no.
			Gellan	Welan	Diutan	
***Biosynthesis of nucleotide*****-*****sugar precursors***						
***manB*****/*****pgmG***	460	Phosphoglucomutase/phosphomannomutase	80.3 (87.7)	80.1 (87.2)	—	AHE56248.1
***manA***	277	Mannose-6-phosphate isomerase	71.7 (80.4)	71.5 (80.4)	—	AHE57091.1
***manC***	342	Mannose-1-phosphate guanylyltransferase	54.8 (66.7)	63.2 (76.8)	—	AHE56266.1
***ugpG***	288	Glucose- 1-phosphate uridylyltransferase	76.2 (86.1)	74.8 (86.9)	—	AHE52897.1
***ugdG***	454	UDP-glucose-6-dehydrogenase	70.8 (80.4)	70.5 (80.4)	—	AHE52667.1
***rmlA***	288	Glucose-1-phosphate thymidylyltransferase	60.3 (77.4)	60.6 (76.4)	61.6 (77.7)	AHE57212.1
***rmlB***	356	dTDP-glucose 4,6-dehydratase	63.1 (74.3)	63.4 (74.6)	63.1 (74.3)	AHE57214.1
***rmlC***	186	dTDP-4-dehydrorhamnose 3,5-epimerase	48.2 (62.6)	49.2 (62.6)	48.2 (63.1)	AHE57215.1
***rmlD***	297	dTDP-4-dehydrorhamnose reductase	43.4 (56.2)	42.8 (54.9)	42.1 (55.2)	AHE57213.1
***Assembly of the repeat units***						
***ssB***	468	Glucosyl-isoprenylphosphate transferase	41.6 (57.4)	43.4 (69.6)	44.1 (60.1)	AHE57216.1
***ssQ***	327	Rhamnosyl transferase	22.6 (34.7)	12.5 (17.1)	21.1 (36.6)	AHE56442.1
***ssH***	428	Glycosyl transferase	N	N	—	AHE52671.1
***ssT***	289	Glycosyl transferase	N	N	—	AHE56448.1
***ssX***	652	Acyltransferase	N	N	—	AHE56441.1
***ssY***	352	Acyltransferase	N	N	—	AHE56447.1
***ssZ***	372	Acyltransferase	N	N	—	AHE57208.1
***ssI***	521	Alginate O-acetylation protein	N	N	—	AHE57204.1
***Polymerization and export of the repeat units***						
***ssC*****-*****N***	1-489/751	Export protein (chain length determinant)	15.6 (29.9)	17.6 (32.3)	16.6 (30.9)	AHE52342.1
***ssC*****-*****C***	490-751/751	Export protein (tyrosine kinase)	20.1 (34.1)	22.8 (36.9)	22.1 (35.9)	AHE52342.1
***ssD***	199	Polysaccharide export protein	19.5 (26.9)	17.7 (27.8)	17.1 (25.1)	AHE52343.1
***ssG***	459	Polysaccharide polymerase	17.5 (27.3)	21.9 (34.7)	20.2 (33.6)	AHE52344.1
***ssS***	482	Polysaccharide biosynthesis protein flippase	14.7 (29.0)	18.7 (33.1)	16.7 (28.5)	AHE57210.1

N: not detected; “—” there is no corresponding genome database.

**Table 2:** Bacterial strains and plasmids used in this study.

Strain or plasmid	Genotype or phenotype	Source or reference
**Strains**		
*E. coli* S17	*RecA thi pro hsdR*^*−*^ M^+^ RP4, Sm^R^ Amp^R^ Kan^R^	This work
*S. sanxanigenens* NX02	Wild-type strain, Ss^+^, Cm^R^	This work
*S. elodea* ATCC31461	Wild-type strain, Gel^+^	This work
*Sphingomonas sp*. ATCC31555	Wild-type strain, Wel^+^	This work
**Plasmids**		
pLO3	4937-bp suicide vector, tet^R^	53
pBBR1MCS-2	5144-bp broad host range vector, kan^R^	54
pLO3ssn	pLO3 derivative carrying upstream and downstream fragment of *ssn*, “*ssn*” refers to all gene related to Ss biosynthesis	This work
pBBRssB	pBBR1MCS-2 derivative expressing *ssB*	This work
pBBRssn	pBBR1MCS-2 derivative expressing *ssn*, “*ssn*” refers to all related genes	This work
pBBRSrssn	pBBR1MCS-2 derivative expressing *ssB and ssn* simultaneously	This work
pBBRSrgeln	pBBR1MCS-2 derivative expressing *ssB and geln* simultaneously, “*geln*” refers to *gelQ, gelL*	This work
pBBRgeln	pBBR1MCS-2 derivative expressing *geln*, “*geln*” refers to *gelD/gelCE/gelS/gelG*	This work
pBBRweln	pBBR1MCS-2 derivative expressing *weln*, “*weln*” refers to *welD/welCE/welS/welG*	This work
