# Supplementary material for: The evolutionary life cycle of the polysaccharide biosynthetic gene cluster based on the Sphingomonadaceae
Source: Sci Rep. 2017 Apr 21;7:46484. doi: 10.1038/srep46484 (PMC5399355; doi:10.1038/srep46484)
Supplement: Supplementary Information [file srep46484-s1.doc]

**Supplementary information**

**Title:** The evolutionary life cycle of the polysaccharide biosynthetic gene cluster based on the *Sphingomonadaceae*

**Authors**

Mengmeng Wu^1†^, Haidong Huang^2†^, Guoqiang Li^1^, Yi Ren^3^, Zhong Shi^1^, Xiaoyan Li^2^, Xiaohui Dai^1^, Ge Gao^1^, Mengnan Ren^2^, Ting Ma^1^*

**Affiliations**

^1^ Key Laboratory of Molecular Microbiology and Technology, Ministry of Education, College of Life Sciences, Nankai University, Tianjin, China

^2^ College of Agronomy & Resources and Environment, Tianjin Agricultural University, Tianjin, China

^3^ Shanghai Majorbio Bio-pharm Biotechnology Limited Company, Shanghai, China

***Corresponding author**

Key Laboratory of Molecular Microbiology and Technology, Ministry of Education, College of Life Sciences, Nankai University, Tianjin, China

E-mail: [tingma@nankai.edu.cn.](mailto:tingma@nankai.edu.cn.(TM))

^†^ These authors contributed equally to this work.

**Supplementary discussion**

**Genomic islands and gene transfer**

Gene transfer, especially horizontal gene transfer (HGT), is a universally efficient way for microorganisms to acquire functions that enable them to adapt to environments with different selective pressures^66^. In all, 327 insertion sequences (ISs), representing 93.8 kb, were annotated in the NX02 genome, 72 of which are on the plasmid and 255 are on the chromosome (Supplementary Table S1). These ISs were classified into 16 families, according to the ISfinder database, 54 of them occurred in two to six copies. The most frequently identified were ISMdi7, which originated from *Methylobacterium dichloromethanicum,* and ISMex8 from *Methylobacterium extorquens*.

Genomic islands (GIs) in prokaryotic genomes are clusters of genes that are believed to have horizontal origins. There are 43 GIs predicted in the genome of NX02 using the SGIHMM method (Fig. 2). In these 43 GIs, comprising 298,902 bp, 308 CDS were identified, including transposases, transcriptional regulators, and membrane proteins (Supplementary Table S3). For example, one gene island (403,947–418,179 bp) contains genes encoding 6-phosphofructokinase (NX02_01925, with a 92% maximum identity with that of *Novosphingobium pentaromativorans* US6-1), multi-sensor signal transduction histidine kinase (NX02_01955, with a 96% maximum identity with that of *N. pentaromativorans* US6-1), and a transcriptional regulator of the LuxR family (NX02_01960, with a 98% maximum identity with that of *N. pentaromativorans* US6-1). The high identity of these predicted genes with those from the same strain suggested their potential origins from horizontal gene transfer from *N. pentaromativorans* US6-1. A second gene island (489,078–496,868 bp) contains long-chain fatty acid ligase (NX02_02380, with a 80% maximum identity with that of *Novosphingobium nitrogenifigens* DSM 19370) and a membrane protein involved in aromatic hydrocarbon degradation (NX02_02385, with a 79% maximum identity with that of *N. pentaromativorans* US6-1). Another gene island (513,112–522,578 bp) contains genes encoding aldehyde dehydrogenase and alcohol dehydrogenase responding to [alkane](javascript:void(0);) degradation (NX02_02480 and NX02_02610, with 83% and 73% maximum identities with those of *Rhodobacter sp.* SW2 and *Oceanicaulis alexandrii* HTCC2633, respectively). Lastly, a gene island (1,879,905–1,924,127 bp) was identified that contains genes encoding heat shock protein Hsp20 (NX02_09300, with a 75% maximum identity with that of *Sphingobium chlorophenolicum* L-1) and aminoglycoside phosphotransferase (NX02_09305, with a 74% maximum identity with that of *N. pentaromativorans* US6-1). The identity of these predicted genes with those from different bacteria might suggest their potential origins from horizontal gene transfer, enabling the bacterium to adapt to the alkane degradation and adverse environmental impact.

**Secretion systems**

Gram-negative bacteria are surrounded by a dual membrane structure establishing an interface between the environment and the interior of the cells. The two membranes are separated by an aqueous periplasmic space containing a rigid peptidoglycan layer. This cell envelope constitutes a highly selective barrier for the uptake and release of various compounds^67^. Complex interactions between Gram-negative bacteria and their environment are facilitated by numerous surface-attached and exported macromolecules. To cross the two bacterial membranes, those molecules are transported by secretion systems^68^. These secretion systems were classified into six types: from type I through type VI secretion system (T1SS-T6SS). *S. elodea* ATCC 31461, *Sphingomonas sp*. ATCC 31555, and *S. wittichii* RW1 all contain one set of T1SS, T2SS and T4SS. *Sphingomonas sp*. ATCC 31555 also has one T3SS. The genome of *S. sanxanigenens* NX02 is bigger than the other three strains, and contains a wide variety of secretion systems, including one T1SS, one T2SS, one T3SS, four T4SSs, and one T6SS (Supplementary Fig. S2).

T1SS is required for the secretion of a variety of degradative enzymes and offensive molecules. These include a variety of hydrolases and proteinaceous toxins^69^. The relatively simple exporter complex comprises only three protein subunits: an inner membrane-bound ATP-binding cassette (ABC) protein that forms a complex with a membrane fusion protein (MFP) in the periplasmic space, and an outer membrane protein (OMP) that resides in the periplasmic space and is embedded in the outer membrane. T1SS is best represented by the *Escherichia coli* hemolysin (Hly) secretion system, which consists of HlyB as the ABC protein, HlyD as the MFP, and TolC as the OMP. The gene encoding TolC is usually not found within the Hly operon^70^. A T1SS of NX02 comprised of ABC-MFP and OMP genes within different operon. The TolC protein shows 71% similarity to the outer membrane protein of *Sphingomonas sp. SKA58*. TolC is a multifunctional protein involved in many important cellular processes, probably the most important one being multidrug efflux. Twelve *gsp* genes, including genes denoted *gspC* to *gspO*, are found in the genome of NX02. *E. coli* and other bacteria have a variety of extrusion mechanisms to eliminate such xenobiotics. The transport substrates and T1SS component ABC-MFP are encoded by gene clusters found integrated into the NX02 chromosome. Three ABC-MFP genes were identified in the genome of NX02; the MFP genes are highly similar (≥73%) to *hlyD* of *Sphingomonas sp. SKA58*; however, ABC genes do not show close homology with *hlyB*. Two ABC-MFP genes are adjacent to transport substrates outer membrane lipoprotein of RND (Resistance-Nodulation-cell Division) type efflux systems. The transporter provides drug resistance through a tripartite efflux pump comprising the RND protein, ABC-MFP and OMP.

The T2SS is responsible for the extracellular secretion of toxins and hydrolytic enzymes. Proteins secreted through TSS2 depend on the Sec or twin-arginine translocation (TAT) system for initial transport into the periplasm. The genome of *S. sanxanigenens* NX02 encodes a complete Sec secretion system (SecABDEFGY, YajC, FtsY and SRP), and partial proteins of the TAT secretion system (TatBCD). Typical T2SSs are encoded by a set of 12 to 16 *gsp* (general secretion pathway) genes organized into a large operon. Thirteen *gsp* genes, including genes denoted *gspC* to *gspO,* are found in the genome of *S. sanxanigenens* NX02. Six of the 13 conserved *gsp* genes are dedicated to the formation of a periplasmic pilus-like structure called the pseudopilus, a central structure of the T2SS machine. Five of those genes, *gspG*, *H*, *I*, *J* and *K*, encode the pseudopilins which are the constitutive elements of the pseudopilus, whereas *gspO* encodes the prepilin peptidase involved in their maturation^71^. The five pseudopilins are synthesized as precursors with a short leader peptide of 6–7, mostly charged, residues that are cleaved off by the prepilin peptidase GspO. Interestingly, gene *gspO* of NX02 is located in the opposite direction to the operon, close to *gspC*, but not in the *gsp* operon. Moreover, *gspO,* *gspE*, *F*, *L* and *M* genes encode components of the inner membrane platform; the *gspD* gene encodes the secretin; the *gspC* gene encodes the trans-periplasmic protein; and GspC is a component of the inner membrane surface interacting with secretin. The *gspS* or *N* genes are not considered core components of the T2SS. Genes encoding GspS members have not been found in NX02. Its function, stabilization of the pseudopilus, is provided by GspN, just like the T2SS in *Vibrio cholerae* and *Aeromonas hydrophila*^67^. Based on the presence of the complete Sec transport systems and *gsp* operon, we believe the type II secretion system is likely to be functional.

TheT3SS is employed by a number of Gram-negative bacterial pathogens to inject toxins into eukaryotic cells^72^. The T3SS export mechanism usually comprises more than 20 different proteins, including soluble cytoplasmic proteins, outer membrane proteins and integral membrane proteins^73^. The T3SS comprises 11 gene products from *NX02_15795* to *NX02_15875*, which all share higher homology with T3SS components from *Sphingomonas sp. SKA58*. The T3SS genes of NX02 are physically clustered and may have been acquired via horizontal gene transfer as a single “pathogenicity island”. They were identified as a number of cytoplasmic proteins and complete integral membrane proteins, such as YscC, YscJ, YscR, YscS, YscT, YscU, YscV, YscN, YscQ, YscL, and YscD, which comprise integrated T3SS basal body and export apparatus. Some components of the T3SS, including YscF, YscO, YscP, YscX, and YscW, were not found in the genome. It is possible that they can be substituted by type IV pilus proteins encoded in the genome, because they are homologous and functionally equivalent.

The T4SS consists of specialized macromolecule delivery machines that are ancestrally related to bacterial conjugation systems^74^. These systems are broadly classified as type IVA (T4ASS) or IVB (T4BSS), depending on whether their structural components resemble the VirB/D4 complex of the plant pathogen *Agrobacterium tumefaciens* or the conjugal transfer system of the self-transmissible IncI plasmid, respectively^75^. The T4ASS comprises 12 proteins, named VirB1 to VirB11 and VirD4, while the Dot/Icm T4BSS requires up to 27 proteins for efficient function. Four sets of T4SSs are present in NX02, namely T4SS-1 (*NX02_p0495* to *NX02_p0580*), T4SS-2 (*NX02_09735* to *NX02_09825*), T4SS-3 (*NX02_11790* to *NX02_11845*), and T4SS-4 (*NX02_19725* to *NX02_19775*) (Supplementary Fig. S2). The T4SS-1 is found on the plasmid, while T4SS-1, -2, and -3 are on the chromosome. The F sex factor of *E. coli* is a paradigm for bacterial conjugation and its transfer (*tra*) region represents a subset of the T4SS family. An F-like T4SS appears to be involved in the transfer of DNA only, whereas P- and I-type systems appear to transport proteins or nucleoprotein complexes. A putative F-like T4SS operon (T4SS-1, on the plasmid) contains all the components needed to assemble a T4SS complex, including the core T4SS proteins, TraA (pilin), L, E, K, B, V, C, and G (N-terminal domain); also the auxiliary gene products TraF, G (C-terminal domain), H, N, U, and W; and TrbC for pilus assembly and mating pair stabilization. Except TraE, the most homologous proteins were orthologs from *Sphingomonas sp. S17*. Other non-essential components of F-like T4SSs were also found in the operon, such as TrbI, a protein that promotes DNA transport, and TraH, which are localized to the periplasm/outer membrane. Another F-like T4SS-2 on the chromosome encodes 12 proteins, but show higher identity to proteins from *Novosphingobium sp*. PP1Y and *Sphingobium japonicum* UT26S. The two F-like T4SSs may have been acquired and evolved independently. P-type T4SS was exemplified by the IncP plasmid RP4, and includes VirB1–VirB11 and VirD. Both T4SS-3 and T4SS-4 belong to P-type T4SS group. The two T4SSs do not share either the same genetic organization or high sequence identity at the protein level. T4SS-3 has higher identity with the proteins of *Ketogulonicigenium vulgare* Y25 and *Zymomonas mobilis subsp. mobilis* NCIMB 11163. The products of T4SS-4 show higher identity with proteins of *Sphingobium sp. SYK-6* and *Sphingomonas sp. KC8*. Each P-type T4SS contained VirD4, VirB2-5, 9-11, TrbL, J, and a CopG family transcriptional regulator, whereas the T4SS-3 carries a TrbK. Proteins VirB6–8 were not found in *S. sanxanigenens* NX02.

The T6SS is an organelle that is structurally and mechanistically analogous to an intracellular membrane-attached contractile phage tail. It was defined functionally in 2006 in *Vibrio cholerae* through genetic identification of several of its critical components and canonical substrates^76^. The T6SS secretion machinery is the product of approximately 15 conserved genes that are generally found together at a genomic locus^77^. T6SSs have since been identified in the genomes of several pathogenic and nonpathogenic Gram-negative bacteria, suggesting that it may be involved in functions unrelated to pathogenicity^78^. The T6SS of *S. sanxanigenens* NX02 comprises 15 genes in the *imp* operon, including VgrG, Hcp, VasU, and ClpV. DNA-binding transcriptional regulator AraC was also found in the operon. Interestingly, 12 genes of NX02 T6SS are most similar to *imp* proteins in *Sphingomonas sp.* S17. Other genes in the same operon encode proteins that show high identity with proteins from different strains, such as *Flavobacterium johnsoniae* UW101 and *Agrobacterium radiobacter* K84.

**The identification of the biosynthetic pathway of the biopolymer sanxan**

**Genes and enzymes involved in the formation of nucleotide sugar precursors.** The amino acid of *NX02_14005* showed the highest identity with protein (80%, WP 048937182.1) from *Sphingobium yanoikuyae* and *NX02_23160*, encoding a phosphomannomutase/phosphoglucomutase, its product showed the highest identity with protein (80%, WP_010545852.1) from *S. elodea*. The gene *ugpG*, showed the highest identity with protein (81%, WP 025561166.1) form *Sphingomonas* sp. UNC305MFCol5.2. Protein RmlA showed the identity with protein (76%, WP 037556970.1) from *Sphingopyxis.* Sp. LC363. Protein RmlB was 78% identity with protein (WP 046349117.1) from *S. changbaiensis*. Protein RmlC showed the highest identity with protein (68%, WP 046349116.1) from *S. changbaiensis* and Protein RmlD was 56% identity with protein (WP 046408153.1) from *Sphingomonas.* sp. Ag1. These four genes were clustered and were arranged as the sequence *rmlC‒B‒D‒A*, while those in *S. elodea*, *Sphingomonas.* sp. ATCC31555, and other sphingan-producing strains were all *rmlA‒C‒B‒D*^11^.

There were four genes could encode the protein ManA: *NX02_24810*, *NX02_27530*, *NX02_P0360*, and *NX02_P1145*. Except *NX02_P0360*, other three genes all showed higher FPKM values. *NX02_24810* was located in a gene island area and its deficient strain had an Ss^+^ phenotype, while *NX02_27530* deleted strain had an Ss^-^ phenotype (figure not shown), and it would be complemented by pBBR27530. The remaining two genes were located in the plasmid and their deletions were insignificant. These results suggested that ManA was NX02_27530 (66% dentity with protein from *Sphingomonas* sp. 37zxx (WP 033923207.1)). Protein ManB was encoded by the bifunctional *orf4255* gene, which was also *pgmG* gene. *NX02_23250*, the product was ManC, showed 73% identity with enzyme from *Sphingomonas* sp. RIT328 (EZP54770.1), its’ mutant also had an Ss^-^ phenotype. NX02_p0235, located in the plasmid, was also annotated as Mannose-1-phosphate guanylyltransferase, but its FPKM value was low (43.03).

**Genes and enzymes involved in the assembly, polymerization and export of repeat units.** According to CAZY database and gene annotation, 33 GTs were found in the chromosome. Their FPKM values under sanxan producible condition are shown in Supplementary Fig. S3. The expression levels of the most GTs were also validated by qRT-PCR (data not shown). Besides *ssB*, the FPKM value of *NX02_01520*, *NX02_01730*, *NX02_04645*, *NX02_05090*, *NX02_10600*, *NX02_21065*, *NX02_24170*, *NX02_24200*, *NX02_24770*, *NX02_24825*, *NX02_24830*, *NX02_25225*, and *NX02_27225* were all higher than 100 (Supplementary Fig. S3). Among of them, *NX02_24770*, *NX02_24825*, and *NX02_24830* were located in a gene island, and their deletion strains all had an Ss^+^ phenotype. Although the FPKM values of *NX02_01730*, *NX02_21065*, and *NX02_25225* were high, their deletion could not affect the production of sanxan (Supplementary Fig. S3). Through the observation of phenotypes of mutants and the comparison of sanxan yield of different strains, the disruption of *NX02_10600*, *NX02_01520*, *NX02_05090*, and *NX02_27225* were hardly relevant with sanxan biosynthesis. Only *NX02_24170*, *NX02_4200*, and *NX02_04645* were closely related to sanxan biosynthesis.

Besides the markless knockout of the high-FPKM value genes, homology analysis of genes from *gel*, *wel*, *dps*, or *sps* clusters with those from NX02 was performed with programme tBlastn in Bioedit software. From homology analysis, *NX02_14445* (FPKM 52.52), *NX02_19250* (FPKM 1.79), and *NX02_* 25735 (FPKM 93.61) showed the highest identity with gene *gelS/welS*, *dpsS*, and *spsS*, respectively, but the mutants in *NX02_14445* was lethal both in wild-type and NX02 (∆*ssB*) strains, *NX02_19250* and *NX02_* 25735 knockout strains produced the same amount sanxan as wild-type strain. Although protein SsS encoded by *orf5186* showed low 14.7%, 18.7%, and 16.7% identity with GelS, WelS, and DpsS, respectively, it showed the highest identity with RfbX protein(40%), a membrane protein involved in the export of O-antigen and teichoic acid from *Methylobacterium nodulans* based on NCBI database. Protein SsS was an integral membrane protein with ten predicted transmembrane segments.

**Reference**

1. Siddaramappa, S. *et al*. Horizontal gene transfer in Histophilus somni and its role in the evolution of pathogenic strain 2336, as determined by comparative genomic analyses. *BMC Genomics* **12,** 570 (2011).
2. Douzi, B., Filloux, A. & Voulhoux, R. On the path to uncover the bacterial type II secretion system. *Phil. Trans. R. Soc. B.* **367,** 1059-1072 (2012).
3. Boyer, F., Fichant, G., Berthod, J., Vandenbrouck, Y. & Attree, I. Dissecting the bacterial type VI secretion system by a genome wide in silico analysis: what can be learned from available microbial genomic resources? *BMC Genomics* **10,** 104 (2009).
4. Pieretti, I. *et al*. Genomic insights into strategies used by *Xanthomonas albilineans* with its reduced artillery to spread within sugarcane xylem vessels. *BMC Genomics* **13,** 658 (2012).
5. Thomas, S., Holland, I. B. & Schmitt, L. The Type 1 secretion pathway - the hemolysin system and beyond. *BBA-Mol. Cell Res.* 1843, 1629-1641 (2014).
6. Nunn, D. Bacterial type II protein export and pilus biogenesis: more than just homologies? *Trends Cell Biol.* **9,** 402-408 (1999).
7. Abrusci, P., McDowell, M. A., Lea, S. M. & Johnson, S. Building a secreting nanomachine: a structural overview of the T3SS. *Curr. Opin. Struc. Biol.* 25, 111-117 (2014).
8. Izore, T., Job, V. & Dessen, A. Biogenesis, regulation, and targeting of the type III secretion system. *Structure* 19, 603-612 (2011).
9. Holland, I. B, Schmitt, L. & Young, J. Type 1 protein secretion in bacteria, the ABC-transporter dependent pathway (review). *Mol. Membr. Biol.* 22, 29-39 (2005).
10. Zuleta, L. F. *et al*. The complete genome of *Burkholderia phenoliruptrix* strain BR3459a, a symbiont of Mimosa flocculosa: highlighting the coexistence of symbiotic and pathogenic genes. *BMC Genomics* 15, 535 (2014).
11. Pukatzki, S. *et al*. Identification of a conserved bacterial protein secretion system in *Vibrio cholerae* using the Dictyostelium host model system. *P. Natl. Acad. Sci. USA* 103, 1528-1533 (2006).
12. Redondo-Nieto, M. *et al*. Genome sequence reveals that *Pseudomonas fluorescens* F113 possesses a large and diverse array of systems for rhizosphere function and host interaction. *BMC Genomics* 14, 54 (2013)
13. Shyntum, D. Y., Venter, S. N., Moleleki, L. N., Toth, I. & Coutinho, T. A. Comparative genomics of type VI secretion systems in strains of *Pantoea ananatis* from different environments. *BMC Genomics* 15, 163 (2014).

**Table S1.** Insert sequences in the genome of *S. sanxanigenens* NX02 (E value < 1.00^e-20^)

| **Start** | **End** | **Size (bp)** | **Name** | **e-value** | **Score** | **Identical** |
| --- | --- | --- | --- | --- | --- | --- |
| Chromosome | | | | | | |
| 369231 | 369534 | 304 | ISSsp4 | 3.00^e-73^ | 144 | 87% |
| 369359 | 369480 | 124 | ISMex39 | 6.00^e-22^ | 58 | 88% |
| 370529 | 371090 | 563 | ISSsp4 | 3.00^e-58^ | 119 | 81% |
| 370839 | 371077 | 242 | ISMdi27 | 2.00^e-25^ | 64 | 82% |
| 370851 | 371077 | 230 | ISMex39 | 7.00^e-28^ | 68 | 83% |
| 370895 | 371077 | 183 | ISPkr1 | 7.00^e-25^ | 63 | 84% |
| 436098 | 436315 | 218 | ISRm5 | 8.00^e-34^ | 78 | 84% |
| 436649 | 437083 | 435 | ISRm5 | 9.00^e-49^ | 103 | 81% |
| 441353 | 442059 | 707 | ISMex8 | 1.00^e-127^ | 235 | 83% |
| 441354 | 442059 | 706 | ISMdi7 | 1.00^e-110^ | 206 | 82% |
| 442318 | 442696 | 379 | ISMex8 | 8.00^e-37^ | 83 | 80% |
| 442318 | 442529 | 212 | ISMdi7 | 4.00^e-23^ | 60 | 82% |
| 442558 | 442696 | 139 | ISMdi7 | 1.00^e-29^ | 71 | 88% |
| 442855 | 443249 | 395 | ISMex8 | 6.00^e-56^ | 115 | 82% |
| 442867 | 443348 | 482 | ISMdi7 | 2.00^e-62^ | 126 | 82% |
| 443271 | 443486 | 216 | ISMex8 | 5.00^e-35^ | 80 | 84% |
| 450772 | 450989 | 218 | ISRm5 | 8.00^e-34^ | 78 | 84% |
| 451323 | 451757 | 435 | ISRm5 | 9.00^e-49^ | 103 | 81% |
| 489014 | 489444 | 435 | ISPfe1 | 1.00^e-35^ | 81 | 80% |
| 489064 | 489223 | 160 | ISOba2 | 3.00^e-30^ | 72 | 86% |
| 489064 | 489226 | 163 | ISRssp1 | 3.00^e-27^ | 67 | 85% |
| 489255 | 489582 | 328 | ISOba2 | 2.00^e-37^ | 84 | 81% |
| 489255 | 489582 | 328 | ISRssp1 | 4.00^e-23^ | 60 | 80% |
| 489680 | 490090 | 411 | ISRosp2 | 2.00^e-34^ | 79 | 80% |
| 489731 | 490090 | 360 | ISPfe1 | 7.00^e-28^ | 68 | 80% |
| 517863 | 518302 | 440 | ISMpo10 | 2.00^e-25^ | 64 | 79% |
| 543811 | 544029 | 219 | ISXau4 | 7.00^e-25^ | 63 | 82% |
| 551958 | 552072 | 115 | ISAzs28 | 2.00^e-22^ | 59 | 88% |
| 552125 | 552569 | 445 | ISAzs28 | 2.00^e-83^ | 161 | 84% |
| 588597 | 588900 | 304 | ISSsp4 | 3.00^e-73^ | 144 | 87% |
| 588725 | 588846 | 124 | ISMex39 | 6.00^e-22^ | 58 | 88% |
| 589895 | 590456 | 563 | ISSsp4 | 3.00^e-58^ | 119 | 81% |
| 590205 | 590443 | 242 | ISMdi27 | 2.00^e-25^ | 64 | 82% |
| 590217 | 590443 | 230 | ISMex39 | 7.00^e-28^ | 68 | 83% |
| 590261 | 590443 | 183 | ISPkr1 | 7.00^e-25^ | 63 | 84% |
| 662289 | 662710 | 422 | ISAr1 | 1.00^e-69^ | 138 | 83% |
| 662289 | 662765 | 478 | IS870 | 2.00^e-55^ | 114 | 81% |
| 662394 | 662894 | 501 | ISRfr1 | 2.00^e-52^ | 109 | 80% |
| 662812 | 663050 | 239 | ISAr1 | 5.00^e-44^ | 95 | 85% |
| 662815 | 663038 | 224 | IS870 | 2.00^e-37^ | 84 | 84% |
| 849694 | 850011 | 318 | ISAzs10 | 8.00^e-34^ | 78 | 81% |
| 1057486 | 1057703 | 218 | ISSsp4 | 1.00^e-38^ | 86 | 85% |
| 1057492 | 1057735 | 247 | ISMdi27 | 3.00^e-33^ | 77 | 83% |
| 1057492 | 1057712 | 221 | ISPkr1 | 7.00^e-31^ | 73 | 83% |
| 1059040 | 1059201 | 162 | ISSsp4 | 1.00^e-26^ | 66 | 85% |
| 1059236 | 1059323 | 88 | ISSsp4 | 1.00^e-20^ | 56 | 91% |
| 1211501 | 1211922 | 422 | ISAr1 | 1.00^e-69^ | 138 | 83% |
| 1211501 | 1211977 | 478 | IS870 | 2.00^e-55^ | 114 | 81% |
| 1211606 | 1212106 | 501 | ISRfr1 | 2.00^e-52^ | 109 | 80% |
| 1212024 | 1212262 | 239 | ISAr1 | 5.00^e-44^ | 95 | 85% |
| 1212027 | 1212250 | 224 | IS870 | 2.00^e-37^ | 84 | 84% |
| 1213094 | 1213411 | 318 | ISAzs10 | 2.00^e-31^ | 74 | 81% |
| 1215419 | 1215637 | 219 | ISXau4 | 7.00^e-25^ | 63 | 82% |
| 1218510 | 1218708 | 199 | ISNwi5 | 3.00^e-27^ | 67 | 83% |
| 1218552 | 1218697 | 146 | ISGdi9 | 4.00^e-29^ | 70 | 87% |
| 1219304 | 1219392 | 89 | ISNwi5 | 4.00^e-26^ | 65 | 93% |
| 1277240 | 1277445 | 206 | ISMtsp13 | 2.00^e-43^ | 94 | 86% |
| 1277278 | 1277469 | 192 | ISRhru4 | 8.00^e-40^ | 88 | 86% |
| 1277298 | 1277519 | 222 | ISPebe1 | 1.00^e-26^ | 66 | 82% |
| 1277306 | 1277784 | 481 | ISRtr1 | 5.00^e-41^ | 90 | 80% |
| 1277564 | 1278114 | 551 | ISRhru4 | 2.00^e-46^ | 99 | 79% |
| 1277689 | 1278060 | 372 | ISMtsp13 | 3.00^e-42^ | 92 | 81% |
| 1277822 | 1278043 | 222 | ISRtr1 | 6.00^e-22^ | 58 | 82% |
| 1277833 | 1278114 | 282 | ISAli6 | 5.00^e-41^ | 90 | 83% |
| 1277849 | 1278055 | 208 | ISPebe1 | 2.00^e-37^ | 84 | 86% |
| 1278169 | 1278466 | 298 | ISMtsp13 | 1.00^e-57^ | 118 | 85% |
| 1278169 | 1278330 | 162 | ISAli6 | 3.00^e-36^ | 82 | 88% |
| 1278169 | 1278330 | 162 | ISRhru4 | 3.00^e-24^ | 62 | 85% |
| 1278170 | 1278336 | 170 | ISRtr1 | 1.00^e-20^ | 56 | 84% |
| 1525137 | 1525843 | 707 | ISMex8 | 1.00^e-127^ | 235 | 83% |
| 1525138 | 1525843 | 706 | ISMdi7 | 1.00^e-110^ | 206 | 82% |
| 1526102 | 1526480 | 379 | ISMex8 | 8.00^e-37^ | 83 | 80% |
| 1526102 | 1526313 | 212 | ISMdi7 | 4.00^e-23^ | 60 | 82% |
| 1526342 | 1526480 | 139 | ISMdi7 | 1.00^e-29^ | 71 | 88% |
| 1526639 | 1527033 | 395 | ISMex8 | 6.00^e-56^ | 115 | 82% |
| 1526651 | 1527132 | 482 | ISMdi7 | 2.00^e-62^ | 126 | 82% |
| 1527055 | 1527270 | 216 | ISMex8 | 5.00^e-35^ | 80 | 84% |
| 1932176 | 1932263 | 88 | ISSsp4 | 1.00^e-20^ | 56 | 91% |
| 1932298 | 1932459 | 162 | ISSsp4 | 1.00^e-26^ | 66 | 85% |
| 1933764 | 1934007 | 247 | ISMdi27 | 3.00^e-33^ | 77 | 83% |
| 1933787 | 1934007 | 221 | ISPkr1 | 7.00^e-31^ | 73 | 83% |
| 1933796 | 1934013 | 218 | ISSsp4 | 1.00^e-38^ | 86 | 85% |
| 1965418 | 1965779 | 362 | IS1313 | 1.00^e-38^ | 86 | 81% |
| 1965531 | 1965789 | 261 | IS692 | 3.00^e-24^ | 62 | 81% |
| 1965732 | 1966138 | 407 | ISRm2 | 2.00^e-65^ | 131 | 83% |
| 1965825 | 1966128 | 304 | IS1313 | 2.00^e-25^ | 64 | 81% |
| 1965894 | 1966069 | 176 | IS692 | 1.00^e-20^ | 56 | 83% |
| 1980457 | 1980914 | 458 | ISAzs28 | 4.00^e-48^ | 102 | 81% |
| 1980976 | 1981080 | 105 | ISAzs28 | 4.00^e-26^ | 65 | 90% |
| 1981170 | 1981334 | 165 | ISAzs28 | 2.00^e-28^ | 69 | 86% |
| 1984331 | 1985559 | 1229 | ISShsp3 | 0 | 512 | 85% |
| 2034743 | 2034958 | 216 | ISMex8 | 5.00^e-35^ | 80 | 84% |
| 2034881 | 2035362 | 482 | ISMdi7 | 2.00^e-62^ | 126 | 82% |
| 2034980 | 2035374 | 395 | ISMex8 | 6.00^e-56^ | 115 | 82% |
| 2035533 | 2035911 | 379 | ISMex8 | 8.00^e-37^ | 83 | 80% |
| 2035533 | 2035671 | 139 | ISMdi7 | 1.00^e-29^ | 71 | 88% |
| 2035700 | 2035911 | 212 | ISMdi7 | 4.00^e-23^ | 60 | 82% |
| 2036170 | 2036876 | 707 | ISMex8 | 1.00^e-127^ | 235 | 83% |
| 2036170 | 2036875 | 706 | ISMdi7 | 1.00^e-110^ | 206 | 82% |
| 2454469 | 2454840 | 372 | ISSpma1 | 1.00^e-149^ | 272 | 93% |
| 2454518 | 2454838 | 321 | ISGbe1 | 1.00^e-66^ | 133 | 85% |
| 2454618 | 2454792 | 175 | ISSpwi1 | 2.00^e-34^ | 79 | 86% |
| 2455573 | 2455660 | 88 | ISSsp4 | 1.00^e-20^ | 56 | 91% |
| 2455695 | 2455856 | 162 | ISSsp4 | 1.00^e-26^ | 66 | 85% |
| 2457161 | 2457404 | 247 | ISMdi27 | 3.00^e-33^ | 77 | 83% |
| 2457184 | 2457404 | 221 | ISPkr1 | 7.00^e-31^ | 73 | 83% |
| 2457193 | 2457410 | 218 | ISSsp4 | 1.00^e-38^ | 86 | 85% |
| 2458367 | 2458602 | 236 | ISMex12 | 4.00^e-23^ | 60 | 81% |
| 2459023 | 2459728 | 706 | ISSpma1 | 0 | 494 | 92% |
| 2459127 | 2459486 | 360 | ISSpwi1 | 3.00^e-61^ | 124 | 84% |
| 2459131 | 2459524 | 394 | ISGbe1 | 4.00^e-60^ | 122 | 83% |
| 2459554 | 2459730 | 177 | ISGbe1 | 6.00^e-50^ | 105 | 90% |
| 2459554 | 2459728 | 175 | ISSpwi1 | 3.00^e-39^ | 87 | 87% |
| 2472110 | 2472376 | 267 | ISMex29 | 2.00^e-34^ | 79 | 82% |
| 2475339 | 2476045 | 707 | ISMex8 | 1.00^e-127^ | 235 | 83% |
| 2475340 | 2476045 | 706 | ISMdi7 | 1.00^e-110^ | 206 | 82% |
| 2476304 | 2476682 | 379 | ISMex8 | 8.00^e-37^ | 83 | 80% |
| 2476304 | 2476515 | 212 | ISMdi7 | 4.00^e-23^ | 60 | 82% |
| 2476544 | 2476682 | 139 | ISMdi7 | 1.00^e-29^ | 71 | 88% |
| 2476841 | 2477235 | 395 | ISMex8 | 6.00^e-56^ | 115 | 82% |
| 2476853 | 2477334 | 482 | ISMdi7 | 2.00^e-62^ | 126 | 82% |
| 2477257 | 2477472 | 216 | ISMex8 | 5.00^e-35^ | 80 | 84% |
| 2479810 | 2479994 | 185 | ISSpma1 | 9.00^e-86^ | 165 | 97% |
| 2495264 | 2495474 | 211 | IS868 | 2.00^e-22^ | 59 | 82% |
| 2495345 | 2495531 | 187 | ISAzsp1 | 1.00^e-29^ | 71 | 84% |
| 2495345 | 2495522 | 178 | ISAzs24 | 1.00^e-26^ | 66 | 84% |
| 2495352 | 2495532 | 181 | ISAli4 | 1.00^e-23^ | 61 | 83% |
| 2495884 | 2496310 | 427 | ISAli4 | 3.00^e-39^ | 87 | 80% |
| 2496043 | 2496302 | 260 | ISMex33 | 4.00^e-23^ | 60 | 81% |
| 2496052 | 2496180 | 129 | IS401 | 3.00^e-21^ | 57 | 86% |
| 2496073 | 2496310 | 238 | ISAzs24 | 3.00^e-36^ | 82 | 84% |
| 2496100 | 2496311 | 212 | ISRtr2 | 1.00^e-32^ | 76 | 84% |
| 2508965 | 2509291 | 327 | ISRm3G | 2.00^e-31^ | 74 | 81% |
| 2508965 | 2509291 | 327 | ISRm3 | 2.00^e-31^ | 74 | 81% |
| 2508965 | 2509249 | 285 | ISNGR8 | 1.00^e-20^ | 56 | 80% |
| 2509015 | 2509291 | 277 | ISRle12 | 3.00^e-21^ | 57 | 81% |
| 2518296 | 2518857 | 563 | ISSsp4 | 3.00^e-58^ | 119 | 81% |
| 2518309 | 2518535 | 230 | ISMex39 | 7.00^e-28^ | 68 | 83% |
| 2518309 | 2518547 | 242 | ISMdi27 | 2.00^e-25^ | 64 | 82% |
| 2518309 | 2518491 | 183 | ISPkr1 | 7.00^e-25^ | 63 | 84% |
| 2519852 | 2520155 | 304 | ISSsp4 | 3.00^e-73^ | 144 | 87% |
| 2519906 | 2520027 | 124 | ISMex39 | 6.00^e-22^ | 58 | 88% |
| 2527219 | 2527301 | 83 | ISXau1 | 2.00^e-22^ | 59 | 93% |
| 2804424 | 2804588 | 165 | ISMdi5 | 1.00^e-23^ | 61 | 84% |
| 2806806 | 2807235 | 430 | ISMdi27 | 8.00^e-77^ | 150 | 84% |
| 2806813 | 2807168 | 356 | ISPkr1 | 9.00^e-52^ | 108 | 83% |
| 2806920 | 2807234 | 315 | ISMex39 | 2.00^e-77^ | 151 | 87% |
| 2807877 | 2808054 | 178 | ISRhru5 | 6.00^e-22^ | 58 | 83% |
| 3128796 | 3129013 | 218 | ISRm5 | 8.00^e-34^ | 78 | 84% |
| 3129347 | 3129781 | 435 | ISRm5 | 9.00^e-49^ | 103 | 81% |
| 3512828 | 3513000 | 173 | ISGdi4 | 4.00^e-26^ | 65 | 84% |
| 3650351 | 3650530 | 180 | ISAzs24 | 3.00^e-30^ | 72 | 85% |
| 3650351 | 3650530 | 180 | ISMex33 | 3.00^e-30^ | 72 | 85% |
| 3650351 | 3650533 | 183 | ISHne1 | 1.00^e-29^ | 71 | 85% |
| 3650353 | 3650533 | 181 | IS868 | 1.00^e-23^ | 61 | 83% |
| 3650365 | 3650527 | 163 | ISDsh1 | 7.00^e-25^ | 63 | 85% |
| 3650374 | 3650530 | 157 | ISAzsp1 | 7.00^e-31^ | 73 | 87% |
| 3650377 | 3650530 | 154 | ISAli4 | 1.00^e-26^ | 66 | 86% |
| 3675689 | 3675846 | 158 | IS427 | 8.00^e-34^ | 78 | 87% |
| 3676068 | 3676467 | 400 | IS427 | 1.00^e-102^ | 192 | 87% |
| 3780114 | 3780411 | 298 | ISMtsp13 | 1.00^e-57^ | 118 | 85% |
| 3780244 | 3780410 | 170 | ISRtr1 | 1.00^e-20^ | 56 | 84% |
| 3780250 | 3780411 | 162 | ISAli6 | 3.00^e-36^ | 82 | 88% |
| 3780250 | 3780411 | 162 | ISRhru4 | 3.00^e-24^ | 62 | 85% |
| 3780466 | 3781016 | 551 | ISRhru4 | 5.00^e-44^ | 95 | 79% |
| 3780466 | 3780747 | 282 | ISAli6 | 5.00^e-41^ | 90 | 83% |
| 3780520 | 3780891 | 372 | ISMtsp13 | 3.00^e-42^ | 92 | 81% |
| 3780525 | 3780731 | 208 | ISPebe1 | 2.00^e-37^ | 84 | 86% |
| 3780537 | 3780758 | 222 | ISRtr1 | 6.00^e-22^ | 58 | 82% |
| 3780895 | 3781274 | 382 | ISRtr1 | 5.00^e-32^ | 75 | 80% |
| 3781061 | 3781195 | 135 | ISPebe1 | 3.00^e-27^ | 67 | 87% |
| 3781117 | 3781292 | 176 | ISRhru4 | 5.00^e-35^ | 80 | 86% |
| 3781135 | 3781340 | 206 | ISMtsp13 | 9.00^e-46^ | 98 | 87% |
| 3781757 | 3781955 | 199 | ISNwi5 | 3.00^e-27^ | 67 | 83% |
| 3781799 | 3781944 | 146 | ISGdi9 | 4.00^e-29^ | 70 | 87% |
| 3782551 | 3782639 | 89 | ISNwi5 | 4.00^e-26^ | 65 | 93% |
| 4079389 | 4079604 | 216 | ISMex8 | 5.00^e-35^ | 80 | 84% |
| 4079527 | 4080008 | 482 | ISMdi7 | 2.00^e-62^ | 126 | 82% |
| 4079626 | 4080020 | 395 | ISMex8 | 6.00^e-56^ | 115 | 82% |
| 4080179 | 4080557 | 379 | ISMex8 | 8.00^e-37^ | 83 | 80% |
| 4080179 | 4080317 | 139 | ISMdi7 | 1.00^e-29^ | 71 | 88% |
| 4080346 | 4080557 | 212 | ISMdi7 | 4.00^e-23^ | 60 | 82% |
| 4080816 | 4081522 | 707 | ISMex8 | 1.00^e-127^ | 235 | 83% |
| 4080816 | 4081521 | 706 | ISMdi7 | 1.00^e-110^ | 206 | 82% |
| 4155367 | 4155605 | 239 | ISAr1 | 5.00^e-44^ | 95 | 85% |
| 4155379 | 4155602 | 224 | IS870 | 2.00^e-37^ | 84 | 84% |
| 4155523 | 4156023 | 501 | ISRfr1 | 2.00^e-52^ | 109 | 80% |
| 4155652 | 4156128 | 478 | IS870 | 2.00^e-55^ | 114 | 81% |
| 4155707 | 4156128 | 422 | ISAr1 | 1.00^e-69^ | 138 | 83% |
| 4161402 | 4161719 | 318 | ISAzs10 | 1.00^e-38^ | 86 | 82% |
| 4161458 | 4161727 | 270 | ISSpal2 | 6.00^e-22^ | 58 | 80% |
| 4184600 | 4185021 | 422 | ISAr1 | 1.00^e-69^ | 138 | 83% |
| 4184600 | 4185076 | 478 | IS870 | 2.00^e-55^ | 114 | 81% |
| 4184705 | 4185205 | 501 | ISRfr1 | 2.00^e-52^ | 109 | 80% |
| 4185123 | 4185361 | 239 | ISAr1 | 5.00^e-44^ | 95 | 85% |
| 4185126 | 4185349 | 224 | IS870 | 2.00^e-37^ | 84 | 84% |
| 4188741 | 4188946 | 206 | ISMtsp13 | 5.00^e-41^ | 90 | 86% |
| 4188789 | 4188964 | 176 | ISRhru4 | 5.00^e-35^ | 80 | 86% |
| 4188807 | 4189285 | 481 | ISRtr1 | 1.00^e-38^ | 86 | 80% |
| 4188886 | 4189014 | 129 | ISPebe1 | 4.00^e-26^ | 65 | 88% |
| 4189065 | 4189615 | 551 | ISRhru4 | 4.00^e-51^ | 107 | 80% |
| 4189190 | 4189561 | 372 | ISMtsp13 | 3.00^e-42^ | 92 | 81% |
| 4189323 | 4189544 | 222 | ISRtr1 | 6.00^e-22^ | 58 | 82% |
| 4189334 | 4189615 | 282 | ISAli6 | 5.00^e-41^ | 90 | 83% |
| 4189350 | 4189556 | 208 | ISPebe1 | 2.00^e-37^ | 84 | 86% |
| 4189670 | 4189967 | 298 | ISMtsp13 | 1.00^e-57^ | 118 | 85% |
| 4189670 | 4189831 | 162 | ISAli6 | 3.00^e-36^ | 82 | 88% |
| 4189670 | 4189831 | 162 | ISRhru4 | 3.00^e-24^ | 62 | 85% |
| 4189671 | 4189837 | 170 | ISRtr1 | 1.00^e-20^ | 56 | 84% |
| 4191251 | 4191469 | 219 | ISXau4 | 7.00^e-25^ | 63 | 82% |
| 4251330 | 4252036 | 707 | ISMex8 | 1.00^e-127^ | 235 | 83% |
| 4251331 | 4252036 | 706 | ISMdi7 | 1.00^e-110^ | 206 | 82% |
| 4252295 | 4252673 | 379 | ISMex8 | 8.00^e-37^ | 83 | 80% |
| 4252295 | 4252506 | 212 | ISMdi7 | 4.00^e-23^ | 60 | 82% |
| 4252535 | 4252673 | 139 | ISMdi7 | 1.00^e-29^ | 71 | 88% |
| 4252832 | 4253226 | 395 | ISMex8 | 6.00^e-56^ | 115 | 82% |
| 4252844 | 4253325 | 482 | ISMdi7 | 2.00^e-62^ | 126 | 82% |
| 4253248 | 4253463 | 216 | ISMex8 | 5.00^e-35^ | 80 | 84% |
| 4532392 | 4532607 | 216 | ISAzs17 | 1.00^e-20^ | 56 | 81% |
| 4576268 | 4576503 | 236 | ISMex12 | 4.00^e-23^ | 60 | 81% |
| 5110787 | 5111208 | 422 | ISAr1 | 1.00^e-69^ | 138 | 83% |
| 5110787 | 5111263 | 478 | IS870 | 2.00^e-55^ | 114 | 81% |
| 5110892 | 5111392 | 501 | ISRfr1 | 2.00^e-52^ | 109 | 80% |
| 5111310 | 5111548 | 239 | ISAr1 | 5.00^e-44^ | 95 | 85% |
| 5111313 | 5111536 | 224 | IS870 | 2.00^e-37^ | 84 | 84% |
| 5200708 | 5201005 | 298 | ISMtsp13 | 1.00^e-57^ | 118 | 85% |
| 5200838 | 5201004 | 170 | ISRtr1 | 1.00^e-20^ | 56 | 84% |
| 5200844 | 5201005 | 162 | ISAli6 | 3.00^e-36^ | 82 | 88% |
| 5200844 | 5201005 | 162 | ISRhru4 | 3.00^e-24^ | 62 | 85% |
| 5201060 | 5201610 | 551 | ISRhru4 | 2.00^e-46^ | 99 | 79% |
| 5201060 | 5201341 | 282 | ISAli6 | 5.00^e-41^ | 90 | 83% |
| 5201114 | 5201485 | 372 | ISMtsp13 | 3.00^e-42^ | 92 | 81% |
| 5201119 | 5201325 | 208 | ISPebe1 | 2.00^e-37^ | 84 | 86% |
| 5201131 | 5201352 | 222 | ISRtr1 | 6.00^e-22^ | 58 | 82% |
| 5201390 | 5201868 | 481 | ISRtr1 | 5.00^e-41^ | 90 | 80% |
| 5201655 | 5201876 | 222 | ISPebe1 | 1.00^e-26^ | 66 | 82% |
| 5201705 | 5201896 | 192 | ISRhru4 | 8.00^e-40^ | 88 | 86% |
| 5201729 | 5201934 | 206 | ISMtsp13 | 2.00^e-43^ | 94 | 86% |
| 5220901 | 5221075 | 175 | ISMtsp13 | 2.00^e-22^ | 59 | 83% |
| 5220992 | 5221153 | 162 | ISPebe1 | 6.00^e-22^ | 58 | 84% |
| 5224285 | 5224520 | 236 | ISAli10 | 4.00^e-23^ | 60 | 81% |
| 5224306 | 5224481 | 176 | ISAzs20 | 4.00^e-23^ | 60 | 84% |
| 5496129 | 5496334 | 206 | ISMtsp13 | 2.00^e-43^ | 94 | 86% |
| 5496167 | 5496358 | 192 | ISRhru4 | 8.00^e-40^ | 88 | 86% |
| 5496187 | 5496408 | 222 | ISPebe1 | 1.00^e-26^ | 66 | 82% |
| 5496195 | 5496673 | 481 | ISRtr1 | 5.00^e-41^ | 90 | 80% |
| 5496453 | 5497003 | 551 | ISRhru4 | 2.00^e-46^ | 99 | 79% |
| 5496578 | 5496949 | 372 | ISMtsp13 | 3.00^e-42^ | 92 | 81% |
| 5496711 | 5496932 | 222 | ISRtr1 | 6.00^e-22^ | 58 | 82% |
| 5496722 | 5497003 | 282 | ISAli6 | 5.00^e-41^ | 90 | 83% |
| 5496738 | 5496944 | 208 | ISPebe1 | 2.00^e-37^ | 84 | 86% |
| 5497058 | 5497355 | 298 | ISMtsp13 | 1.00^e-57^ | 118 | 85% |
| 5497058 | 5497219 | 162 | ISAli6 | 3.00^e-36^ | 82 | 88% |
| 5497058 | 5497219 | 162 | ISRhru4 | 3.00^e-24^ | 62 | 85% |
| 5497059 | 5497225 | 170 | ISRtr1 | 1.00^e-20^ | 56 | 84% |

| plasmid | | | | | | |
| --- | --- | --- | --- | --- | --- | --- |
| 21641 | 21731 | 91 | ISSme1 | 4.00^e-26^ | 63 | 92% |
| 21641 | 21731 | 91 | ISRm6 | 4.00^e-26^ | 63 | 92% |
| 21769 | 21978 | 210 | ISEli1 | 2.00^e-25^ | 62 | 82% |
| 21769 | 22127 | 359 | IS511 | 7.00^e-31^ | 71 | 80% |
| 21845 | 21984 | 140 | ISAtu5 | 6.00^e-22^ | 56 | 85% |
| 21860 | 21984 | 125 | ISSme1 | 4.00^e-20^ | 53 | 86% |
| 21860 | 21984 | 125 | ISRm6 | 4.00^e-20^ | 53 | 86% |
| 21862 | 21984 | 123 | ISRm7 | 2.00^e-21^ | 55 | 86% |
| 22596 | 22723 | 128 | ISMex1 | 3.00^e-24^ | 60 | 87% |
| 22778 | 22946 | 169 | ISMex1 | 2.00^e-22^ | 57 | 83% |
| 22778 | 22954 | 177 | ISMamg1 | 6.00^e-25^ | 61 | 84% |
| 22854 | 22946 | 93 | ISPfe1 | 4.00^e-20^ | 53 | 89% |
| 83734 | 83951 | 218 | ISSsp4 | 8.00^e-40^ | 86 | 85% |
| 83740 | 83960 | 221 | ISPkr1 | 4.00^e-32^ | 73 | 83% |
| 83740 | 83971 | 235 | ISMex39 | 4.00^e-20^ | 53 | 81% |
| 83740 | 83983 | 247 | ISMdi27 | 2.00^e-34^ | 77 | 83% |
| 85288 | 85449 | 162 | ISSsp4 | 7.00^e-28^ | 66 | 85% |
| 85343 | 85459 | 117 | ISPkr1 | 4.00^e-20^ | 53 | 86% |
| 85484 | 85571 | 88 | ISSsp4 | 6.00^e-22^ | 56 | 91% |
| 119416 | 119633 | 218 | ISSsp4 | 8.00^e-40^ | 86 | 85% |
| 119422 | 119642 | 221 | ISPkr1 | 4.00^e-32^ | 73 | 83% |
| 119422 | 119653 | 235 | ISMex39 | 4.00^e-20^ | 53 | 81% |
| 119422 | 119665 | 247 | ISMdi27 | 2.00^e-34^ | 77 | 83% |
| 120970 | 121131 | 162 | ISSsp4 | 7.00^e-28^ | 66 | 85% |
| 121025 | 121141 | 117 | ISPkr1 | 4.00^e-20^ | 53 | 86% |
| 121166 | 121253 | 88 | ISSsp4 | 6.00^e-22^ | 56 | 91% |
| 188864 | 189019 | 156 | ISPa42 | 3.00^e-24^ | 60 | 85% |
| 189764 | 189946 | 183 | ISPa42 | 1.00^e-23^ | 59 | 83% |
| 190025 | 190157 | 133 | ISPa42 | 1.00^e-29^ | 69 | 88% |
| 190493 | 190755 | 263 | ISPa42 | 3.00^e-33^ | 75 | 82% |
| 191324 | 191550 | 227 | ISPa42 | 2.00^e-21^ | 55 | 81% |
| 194038 | 194412 | 375 | ISGbe1 | 1.00^e-129^ | 235 | 91% |
| 194044 | 194364 | 321 | ISSpwi1 | 3.00^e-27^ | 65 | 80% |
| 194090 | 194407 | 318 | ISSpma1 | 1.00^e-94^ | 178 | 89% |
| 194200 | 194326 | 127 | ISNha4 | 4.00^e-26^ | 63 | 87% |
| 194821 | 195004 | 184 | ISMex12 | 1.00^e-26^ | 64 | 84% |
| 195395 | 195841 | 447 | ISMex12 | 9.00^e-55^ | 111 | 82% |
| 196077 | 196576 | 500 | ISSpwi1 | 1.00^e-50^ | 104 | 80% |
| 196080 | 196579 | 503 | ISSpma1 | 2.00^e-52^ | 107 | 81% |
| 196083 | 196971 | 889 | ISGbe1 | 0 | 489 | 89% |
| 196596 | 196967 | 372 | ISSpma1 | 3.00^e-67^ | 132 | 84% |
| 196599 | 196887 | 289 | ISSpwi1 | 1.00^e-72^ | 141 | 87% |
| 258238 | 258310 | 73 | ISRtr2 | 4.00^e-20^ | 53 | 93% |
| 258429 | 258639 | 211 | IS868 | 1.00^e-23^ | 59 | 82% |
| 258510 | 258687 | 178 | ISAzs24 | 7.00^e-28^ | 66 | 84% |
| 258510 | 258696 | 187 | ISAzsp1 | 7.00^e-31^ | 71 | 84% |
| 258517 | 258697 | 181 | ISAli4 | 6.00^e-25^ | 61 | 83% |
| 258998 | 259170 | 173 | IS868 | 4.00^e-20^ | 53 | 83% |
| 259049 | 259475 | 427 | ISAli4 | 2.00^e-40^ | 87 | 80% |
| 259208 | 259467 | 260 | ISMex33 | 3.00^e-24^ | 60 | 81% |
| 259217 | 259345 | 129 | IS401 | 2.00^e-22^ | 57 | 86% |
| 259238 | 259475 | 238 | ISAzs24 | 2.00^e-37^ | 82 | 84% |
| 259265 | 259476 | 212 | ISRtr2 | 7.00^e-34^ | 76 | 84% |
| 262930 | 263017 | 88 | ISSsp4 | 6.00^e-22^ | 56 | 91% |
| 263042 | 263158 | 117 | ISPkr1 | 4.00^e-20^ | 53 | 86% |
| 263052 | 263213 | 162 | ISSsp4 | 7.00^e-28^ | 66 | 85% |
| 264518 | 264761 | 247 | ISMdi27 | 2.00^e-34^ | 77 | 83% |
| 264530 | 264761 | 235 | ISMex39 | 4.00^e-20^ | 53 | 81% |
| 264541 | 264761 | 221 | ISPkr1 | 4.00^e-32^ | 73 | 83% |
| 264550 | 264767 | 218 | ISSsp4 | 8.00^e-40^ | 86 | 85% |
| 292030 | 292245 | 216 | ISMex8 | 3.00^e-36^ | 80 | 84% |
| 292168 | 292649 | 482 | ISMdi7 | 1.00^e-63^ | 126 | 82% |
| 292267 | 292661 | 395 | ISMex8 | 4.00^e-57^ | 115 | 82% |
| 292820 | 292958 | 139 | ISMdi7 | 7.00^e-31^ | 71 | 88% |
| 292820 | 293198 | 379 | ISMex8 | 5.00^e-38^ | 83 | 80% |
| 292987 | 293198 | 212 | ISMdi7 | 3.00^e-24^ | 60 | 82% |
| 293457 | 294162 | 706 | ISMdi7 | 1.00^e-111^ | 206 | 82% |
| 293457 | 294163 | 707 | ISMex8 | 1.00^e-129^ | 235 | 83% |
| 358953 | 359125 | 173 | ISAzs17 | 6.00^e-25^ | 61 | 84% |
| 359582 | 359883 | 302 | ISAzs17 | 1.00^e-56^ | 114 | 84% |
| 360008 | 360210 | 203 | ISAzs17 | 8.00^e-43^ | 91 | 86% |
| 360359 | 360546 | 188 | ISAzs17 | 4.00^e-29^ | 68 | 84% |

**Table S2.** Phages in the genome of *S. sanxanigenens* NX02

| **No.** | **Length (kb)** | **Viability** | **CDS** | **Position** | **Specific keyword** | **G+C (%)** |
| --- | --- | --- | --- | --- | --- | --- |
| 1 | 25.9 | incomplete | 38 | 851694~877595 | tail, integrase, protease | 66.27 |
| 2 | 12.9 | incomplete | 16 | 880301~893296 | portal, head, tail | 65.61 |
| 3 | 22.4 | questionable | 14 | 2501167~2523592 | transposase, tail | 63.35 |
| 4 | 8 | incomplete | 13 | 2800763~2808849 | transposase | 64.72 |
| 5 | 28.7 | intact | 35 | 4076698~4105468 | tail, transposase, terminase, portal, head, capsid | 64.91 |
| 6 | 19.8 | questionable | 26 | 4499595~4519468 | terminase, portal, head, tail | 65.37% |
| 7 | 16.7 | questionable | 17 | 4585112~4601874 | tail, head, portal, terminase | 66.06 |
| 8 | 10.9 | incomplete | 21 | 4614837~4625773 | - | 67.49 |
| 9 | 16.4 | questionable | 19 | 6119553~6136007 | terminase, portal, head, tail | 65.89 |
| P10 | 25.3 | incomplete | 17 | p 132594~157945 | - | 65.2 |
| P11 | 8.3 | incomplete | 11 | p 286625~295019 | transposase | 66.09 |
| P12 | 20.3 | incomplete | 7 | p 312792~333121 | transposase | 64.30 |

**Table S3.** Genomic islands prediction by different methods in the genome of *S. sanxanigenens* NX02

| Start | End | Size | GI Prediction Program |
| --- | --- | --- | --- |
| chromosome | | | |
| 276581 | 285541 | 8960 | Predicted by multiple methods |
| 403947 | 418179 | 14232 | Predicted by multiple methods |
| 421699 | 427938 | 6239 | Predicted by multiple methods |
| 467775 | 473379 | 5604 | Predicted by multiple methods |
| 489078 | 496868 | 7790 | Predicted by multiple methods |
| 513112 | 522578 | 9466 | Predicted by multiple methods |
| 538049 | 555330 | 17281 | Predicted by multiple methods |
| 571219 | 576427 | 5208 | Predicted by multiple methods |
| 697368 | 704549 | 7181 | Predicted by multiple methods |
| 836050 | 855039 | 18989 | Predicted by multiple methods |
| 898351 | 904334 | 5983 | Predicted by multiple methods |
| 1054731 | 1064912 | 10181 | Predicted by multiple methods |
| 1073131 | 1080635 | 7504 | Predicted by multiple methods |
| 1180514 | 1190264 | 9750 | Predicted by multiple methods |
| 1195905 | 1222411 | 26506 | Predicted by multiple methods |
| 1247522 | 1253545 | 6023 | Predicted by multiple methods |
| 1319888 | 1326241 | 6353 | Predicted by multiple methods |
| 1492110 | 1496473 | 4363 | Predicted by multiple methods |
| 1879905 | 1884336 | 4431 | Predicted by multiple methods |
| 1919559 | 1924127 | 4568 | Predicted by multiple methods |
| 1929154 | 1947015 | 17861 | Predicted by multiple methods |
| 1954328 | 1959269 | 4941 | Predicted by multiple methods |
| 1961625 | 1990985 | 29360 | Predicted by multiple methods |
| 1999269 | 2004553 | 5284 | Predicted by multiple methods |
| 2018123 | 2036970 | 18847 | Predicted by multiple methods |
| 2063272 | 2076370 | 13098 | Predicted by multiple methods |
| 2221161 | 2228389 | 7228 | Predicted by multiple methods |
| 2450099 | 2481965 | 31866 | Predicted by multiple methods |
| 2497383 | 2517763 | 20380 | Predicted by multiple methods |
| 2520872 | 2526886 | 6014 | Predicted by multiple methods |
| 2572951 | 2578041 | 5090 | Predicted by multiple methods |
| 2804741 | 2808861 | 4120 | Predicted by multiple methods |
| 3510375 | 3518569 | 8194 | Predicted by multiple methods |
| 3669594 | 3678594 | 9000 | Predicted by multiple methods |
| 3998915 | 4003453 | 4538 | Predicted by multiple methods |
| 4066673 | 4080768 | 14095 | Predicted by multiple methods |
| 4111873 | 4121305 | 9432 | Predicted by multiple methods |
| 4156120 | 4161757 | 5637 | Predicted by multiple methods |
| 4182659 | 4214878 | 32219 | Predicted by multiple methods |
| 4491638 | 4497533 | 5895 | Predicted by multiple methods |
| 4525048 | 4531842 | 6794 | Predicted by multiple methods |
| 5200181 | 5215260 | 15079 | Predicted by multiple methods |
| 5311010 | 5327153 | 16143 | Predicted by multiple methods |
| 6106258 | 6112959 | 6701 | Predicted by multiple methods |
| 6142181 | 6147423 | 5242 | Predicted by multiple methods |
| plasmid | | | |
| 259681 | 266050 | 6369 | Predicted by multiple methods |

**Table S4**. Primers used for upstream flank (ssnsu, ssnsl) and downstream flank (ssnxu, ssnxl) amplification, and mutant identification (ssn 1, ssn 2) and complementation (ssn cp1, ssn cp2).

| gene | primers | sequence（5’—3’） | size (bp)  wild-type/mutant |
| --- | --- | --- | --- |
| *ssB* | ssBsu | gccgagctccccaaggtcaagcgcaccatg (*Sac*I) | 1515 |
|  | ssBsl | cggttcttgaggtcggactgatggtgtttgcagttttgtcacgtaag |  |
|  | ssBxu | cttacgtgacaaaactgcaaacaccatcagtccgacctcaagaaccg | 1455 |
|  | ssBxl | tggctctagacccgcgaggcatttgatgatc (*Xba*I) |  |
|  | ssB 1 | agcgatgtgatgcgaaaca | 2010/712 |
|  | ssB 2 | ggtccattggccgtatgtc |  |
|  | ssB cp1 | gactatttctagagcgcgatttaccgttcagtt (*Xba*I) | 1656 |
|  | ssB cp2 | cactatagagctcacgacgacgcggtactgtt (*Sac*I) |  |
| *ssQ* | ssQsu | gagatccttaattaacgcagcccgccttggtaa (*Pac*I) | 1539 |
|  | ssQsl | attgtatcgcccagctcggcccctcgctttatccggttgt |  |
|  | ssQxu | acaaccggataaagcgaggggccgagctgggcgatacaat | 1372 |
|  | ssQxl | gcaggactctagagcgcagacgatcctcgatt (*Xba*I) |  |
|  | ssQ 1 | caggatccgccgctcata | 1300/512 |
|  | ssQ 2 | gcagaacgcgaagagctatctc |  |
|  | ssQ cp1 | aattataggtaccagccggcatgacccatgt (*Kpn*I) | 1012 |
|  | ssQ cp2 | gagtaggaagcttgctcggcgttatgagaatc (*Hin*dⅢ) |  |
| *ssH* | ssHsu | gagataagagctccccttgcggatcgtgattct (*Sac*I) | 1504 |
|  | ssHsl | actgaacgatggccttctccggcaccggcaggttttcc |  |
|  | ssHxu | ggaaaacctgccggtgccggagaaggccatcgttcagt | 1499 |
|  | ssH xl | gctgtcctctagaccgcctatgcagatacgct (*Xba*I) |  |
|  | ssH 1 | ccaaccgcacgatcaaa | 1261/361 |
|  | ssH 2 | gttcgccttcgcatagagc |  |
|  | ssH cp1 | gattataggtaccgaaggaaggctgaccgatg (KpnI) | 1344 |
|  | ssH cp2 | gagataaaagcttcacgggaatgaaaacgcc (*Hin*dⅢ) |  |
| *ssT* | ssTsu | gattatagagctcatgacgatcgcaaggcacacc (*Sac*I) | 542 |
|  | ssTsl | aaccagcgaatgcgtgccaagaaacggtctgtcccaacgc |  |
|  | ssTxu | gcgttgggacagaccgtttcttggcacgcattcgctggtt | 597 |
|  | ssTxl | gtgctagtctagagtcggtagggcgcgaacttcat (*Xba*I) |  |
|  | ssT 1 | cccggtcaaagcgcctagt | 993/291 |
|  | ssT 2 | gcggcgtgctgaaggact |  |
|  | ssT cp1 | gtatctcggtacccaacctcgttcgctgcatc (*Kpn*I) | 849 |
|  | ssT cp2 | gagtacaaagcttgggcgatctgcgatcagtt (*Hin*dⅢ) |  |
| *ssX* | ssXsu | ctatcccttaattaatgcgctacgagaacgagac (*Pac*I) | 1441 |
|  | ssXsl | tcatctcgggctatttgatcaggcggcaatacgagcatt |  |
|  | ssXxu | aatgctcgtattgccgcctgatcaaatagcccgagatga | 1484 |
|  | ssXxl | gagactgtctagagggctatccgctgttcgag (*Xba*I) |  |
|  | ssX 1 | aacgtcaacctgcgctactc | 2268/628 |
|  | ssX 2 | cctcgctttatccggttgtc |  |
|  | ssX cp1 | gatagcaggtaccattgaagtatcgcgcagat (*Kpn*I) | 1984 |
|  | ssX cp2 | gatacataagcttcgatccggcgatcagttc (*Hin*dⅢ) |  |
| *ssY* | ssYsu | gataatagagctccaggcgataggacgtgacc (*Sac*I) | 1502 |
|  | ssYsl | tcttcgtgctctcgggtttggtggaaaagccgttgatg |  |
|  | ssYxu | catcaacggcttttccaccaaacccgagagcacgaaga | 1495 |
|  | ssYxl | gagctagtctagacgtcgatattcccaaccgta (*Xba*I) |  |
|  | ssY 1 | tgcagcgcgtggtcgtg | 1031/251 |
|  | ssY 2 | cgcgacgctggtggtactct |  |
|  | ssY cp1 | gagtagtggtaccggcgatggcgaaacagaag (*Kpn*I) | 1083 |
|  | ssY cp2 | gaggaggtctagacgtgcgctcagccagcta (*Xba*I) |  |
| *ssZ* | ssZsu | gtatatagagctcgctctgcgggattgtcgt (*Sac*I) | 1544 |
|  | ssZsl | tttctaccatgatgacgcggccgccaagctcgtcctc |  |
|  | ssZxu | gaggacgagcttggcggccgcgtcatcatggtagaaa | 1547 |
|  | ssZxl | gagaaggtctagagaagccgtagcgattggtg (*Xba*I) |  |
|  | ssZ 1 | cgttggcgcgatactgttc | 1861/600 |
|  | SsZ 2 | gcttcgggcggtcaatct |  |
|  | ssZ cp1 | ggatatgggtaccttgcgtgaagttcagccaatc (*Kpn*I) | 1148 |
|  | ssZ cp2 | gaggtcaaagcttgaatcaacggtttcatcggg (*Hin*dⅢ) |  |
| *ssI* | ssIsu | gatgcagcttaattaacacatactcgacggtgagg (*Pac*I) | 1510 |
|  | ssIsl | tcatgtcgctgggcttctatcacattgggcatcgtctat |  |
|  | ssIxu | atagacgatgcccaatgtgatagaagcccagcgacatga | 1491 |
|  | ssIxl | gagctactctagaagacgatcgcgcagacctt (*Xba*I) |  |
|  | ssI 1 | ttgcggtggatcgtttcgt | 2032/667 |
|  | ssI 2 | acggcaagctcggataagg |  |
|  | ssI cp1 | ggacgtgggtacctaggatgctgttcaactcgtttg (*Kpn*I) | 1600 |
|  | ssI cp2 | gatcccaaagcttgcttcatcgatgcctccttag (*Hin*dⅢ) |  |
| *ssC* | ssCsu | gataagagagctccgccgttcgacagcttctat (*Sac*I) | 1673 |
|  | ssCsl | caccaagatctatcgctcggccgccatcctgaccaagttc |  |
|  | ssCxu | gaacttggtcaggatggcggccgagcgatagatcttggtg | 1502 |
|  | ssCxl | ctacccatctagagcacgttcgacccctatttc (*Xba*I) |  |
|  | ssC 1 | gttgcggctgagcgacagt | 2775/520 |
|  | ssC 2 | cggccgcaactatctcgttt |  |
|  | ssC cp1 | aatacaaggtacctcaaggtgatctgatgaacga (*Kpn*I) | 2294 |
|  | ssC cp2 | tatatttaagcttcagcggcgggcatcag (*Hin*dⅢ) |  |
| *ssD* | ssDsu | tatgatagagctcgcgatcgccatattgacca (*Sac*I) | 1545 |
|  | ssDsl | ttcgttacagtgtccgtggcacgccaagtctatcgggattt |  |
|  | ssDxu | aaatcccgatagacttggcgtgccacggacactgtaacgaa | 1574 |
|  | ssDxl | gagaacgtctagaggagcagaatggcgacttcc (*Xba*I) |  |
|  | ssD 1 | tggcgagctgcgtgttgag | 1385/725 |
|  | ssD 2 | cgcgctggcagggatattc |  |
|  | ssD cp1 | gatagtcggtaccgtacggtaaaggggaagagat (*Kpn*I) | 756 |
|  | ssD cp2 | gatatgtaagcttttgaacggagcggttgtga (*Hin*dⅢ) |  |
| *ssG* | ssGsu | tggacgcttaattaagaggttgccggtcacatagg (*Pac*I) | 1633 |
|  | ssGsl | agaccggagcagaatggcgaccttcgcagttgcagcatt |  |
|  | ssGxu | aatgctgcaactgcgaaggtcgccattctgctccggtct | 1627 |
|  | ssGxl | gactagctctagacgtgacccggccctatgac (*Xba*I) |  |
|  | ssG 1 | tgccacggacactgtaacga | 1846/550 |
|  | ssG 2 | cggaacagcttcgccttgt |  |
|  | ssG cp1 | caataaaggtaccgagcagaatggcgacttcc (*Kpn*I) | 1409 |
|  | ssG cp2 | gactcgcaagcttaagctctttctacgccattt (*Hin*dⅢ) |  |
| *ssS* | ssSsu | cacgtgattaattaagcaccgcctgtacgaagatg (*Pac*I) | 1206 |
|  | ssSsl | cccacaccttgaagccgtagcgcattgaccaccgcatag |  |
|  | ssSxu | ctatgcggtggtcaatgcgctacggcttcaaggtgtggg | 1213 |
|  | ssSxl | gctgtggtctagactgctgcggaagtgcgtaa (*Xba*I) |  |
|  | ssS 1 | gtgcgaccgttaccagattga | 1779/750 |
|  | ssS 2 | gcattgaccagcccgaacat |  |
|  | ssS cp1 | tatatatggtacctgaacccggcgcaatg (*Kpn*I) | 1477 |
|  | ssS cp2 | gaaataaaagcttgctcattcggccggtt (*Hin*dⅢ) |  |

**Table S5.** Primers used for qRT-PCR

| Primer | Primer sequence (5’-3’) | PCR product size |
| --- | --- | --- |
| 16s q1 | ggcggttcctttagagtaccc | 189 |
| 16s q2 | aaccttaccagcgtttgacatg |
| manB q1 | tatgccgaagccgtgctc | 166 |
| manB q2 | cgccctcttcccagtcg |
| ugpG q1 | gcgatggcgtcgatgat | 162 |
| ugpG q2 | cacctgcttgccgatctc |
| ugdG q1 | aagaccgccgaggatcat | 174 |
| ugdG q2 | catgtcatcggtgttggg |
| manA q1 | gacctcacctatcgcctctacg | 116 |
| manA q2 | gccacgacgacgctcttg |
| manC q1 | tgatgcccagcgaccat | 150 |
| manC q2 | cgccgatcttgatatagcca |
| rmlA q1 | gaggatgccagggcttatg | 196 |
| rmlA q2 | agcggttgagatcggtgat |
| rmlB q1 | gccagggcaccaatgtcc | 106 |
| rmlB q2 | ccgccgaccatgtagctc |
| rmlC q1 | gcgggctgcactatcagg | 160 |
| rmlC q2 | tagagctggctgccgttgg |
| rmlD q1 | cgcacacggcaataatttca | 138 |
| rmlD q2 | gcatcgagcacggcatag |
| ssB q1 | ccgcagatcctgaacgtg | 120 |
| ssB q2 | gcgatcccaataccgatg |
| ssQ q1 | ctgcgtgcggttcggtat | 185 |
| ssQ q2 | caatggcgaacggatggt |
| ssH q1 | aagctatcgccgcatcgc | 153 |
| ssH q2 | acgccgacatagccgacc |
| ssT q1 | gcggtgttcgacaaggtc | 107 |
| ssT q2 | acgtcgtcggcatagacc |
| ssD q1 | tgcggaacacgatcacct | 176 |
| ssD q2 | gcccaaggtctcggtcta |
| ssC q1 | cgcaggcacgttgagcat | 133 |
| ssC q2 | gccgctggtcaatatggc |
| ssG q1 | ctgctgcgaggtgttgtagaa | 105 |
| ssG q2 | ctgtgggtcgtcgtcggt |
| ssS q1 | agcatgagagcggcgaaag | 130 |
| ssS q2 | ccgaagatcagtccgacgat |

**
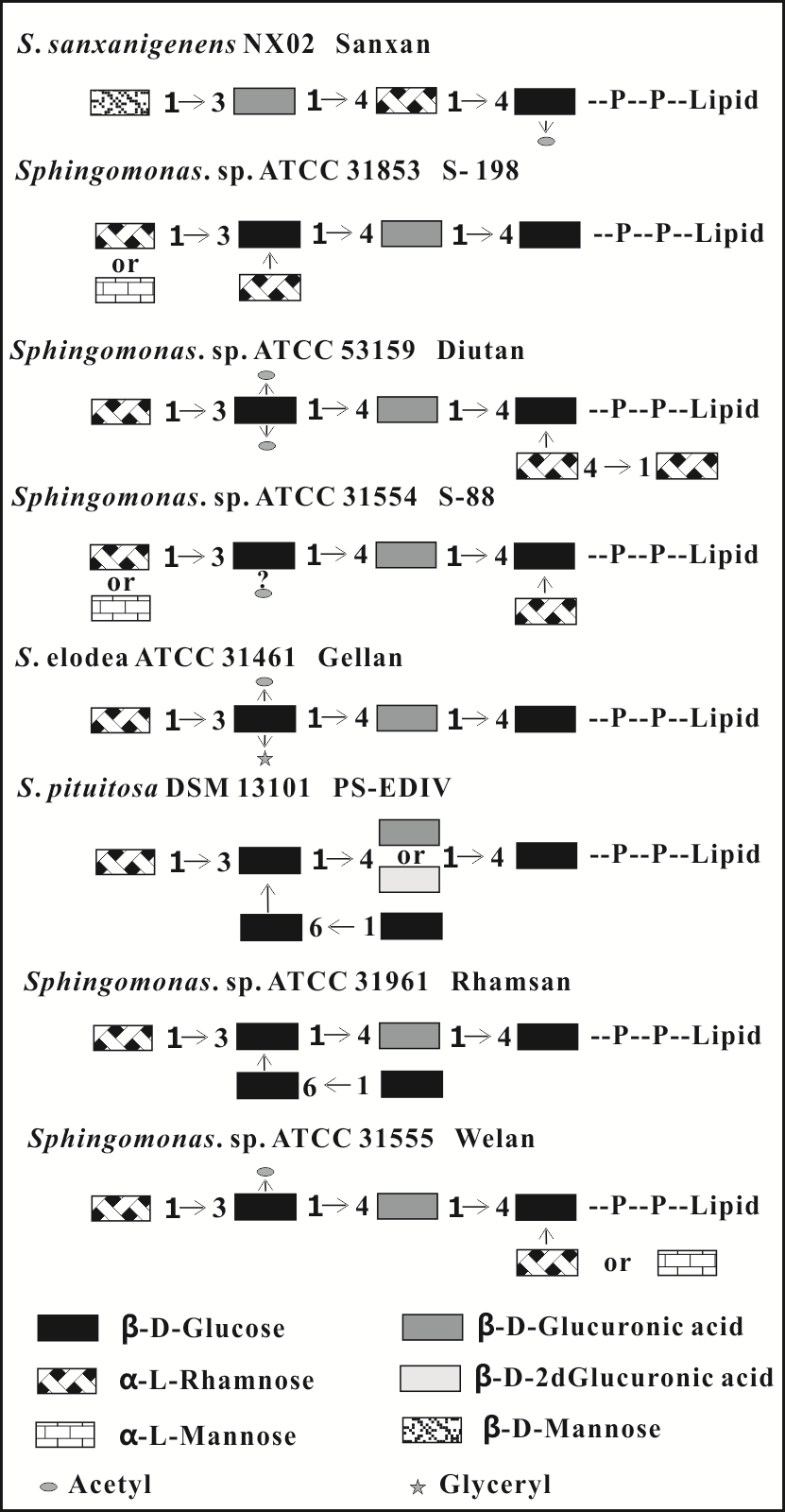
**

**Figure S1.** The repeat structures of sanxan and seven sphingan-producing bacteria.


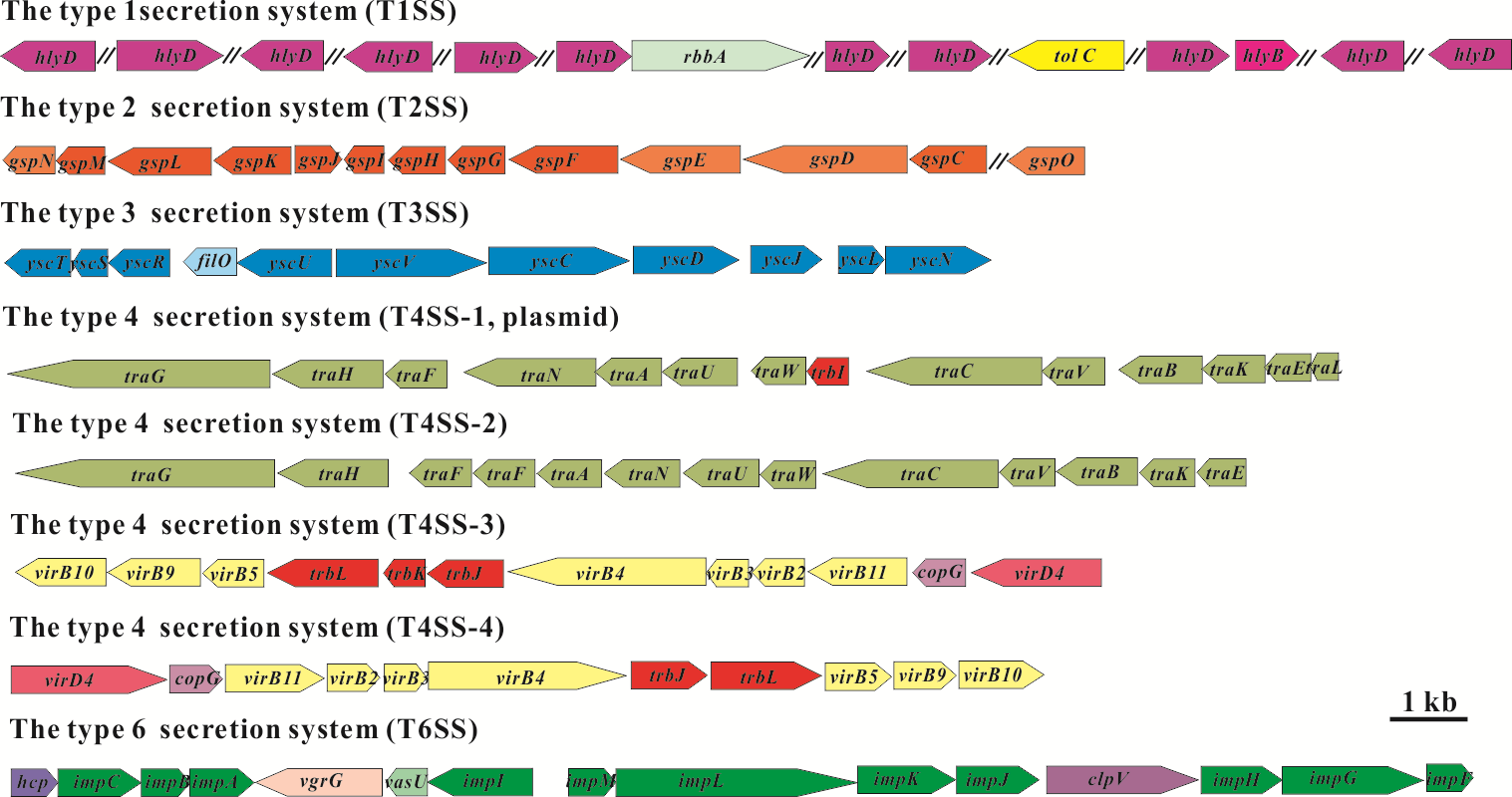


**Figure S2.** Five type secretion systems found in *S. sanxanigenens* NX02 genome (including one T1SS, one T2SS, one T3SS, four T4SSs, and one T6SS).


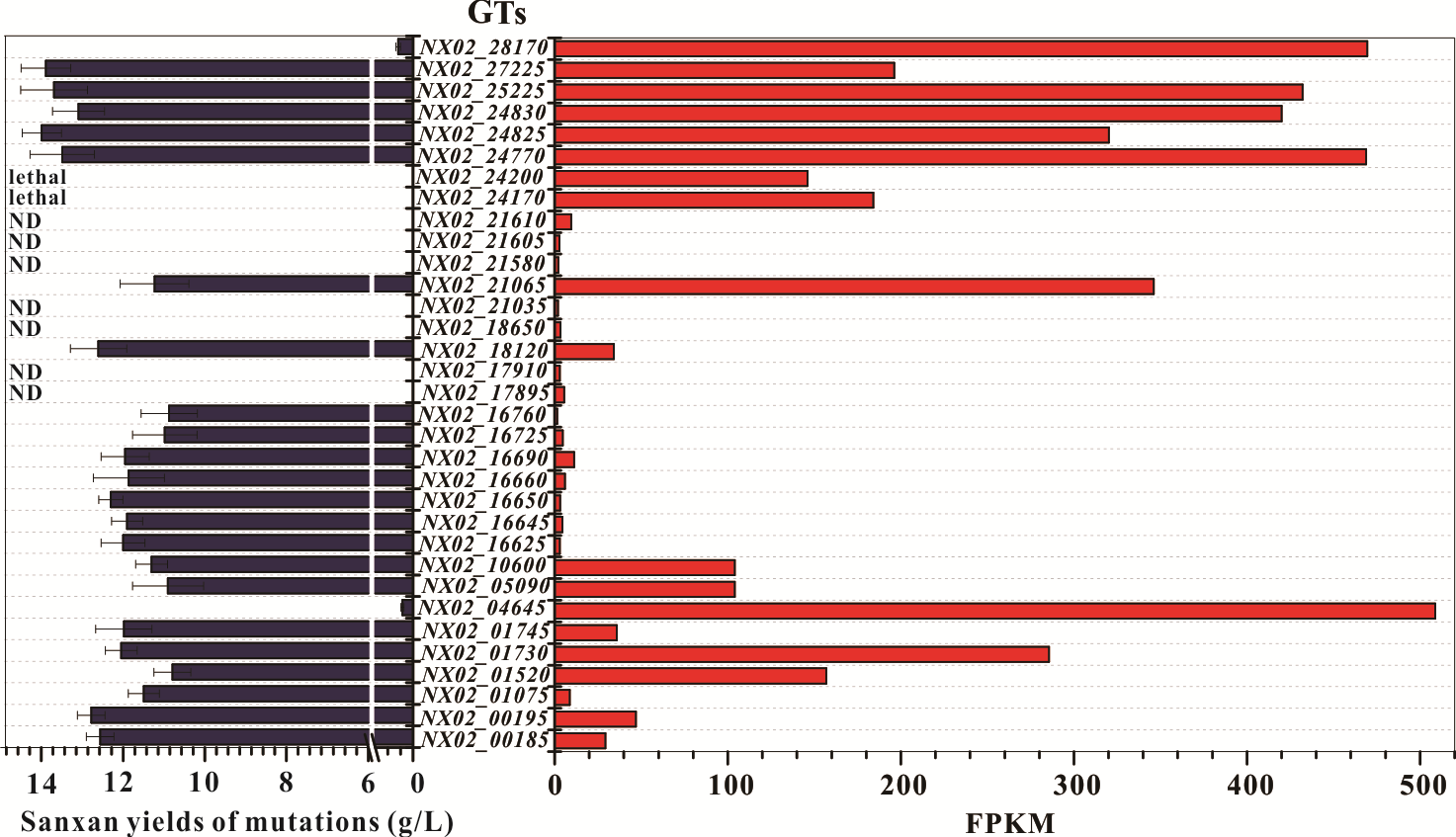


**Figure S3.** FPKM values of 33 glycosyltransferases from NX02 genome, together with sanxan yields of certain mutations. “ND” indicates not detected, “Lethal” means that the mutation of certain gene is lethal in wild-type NX02.

**
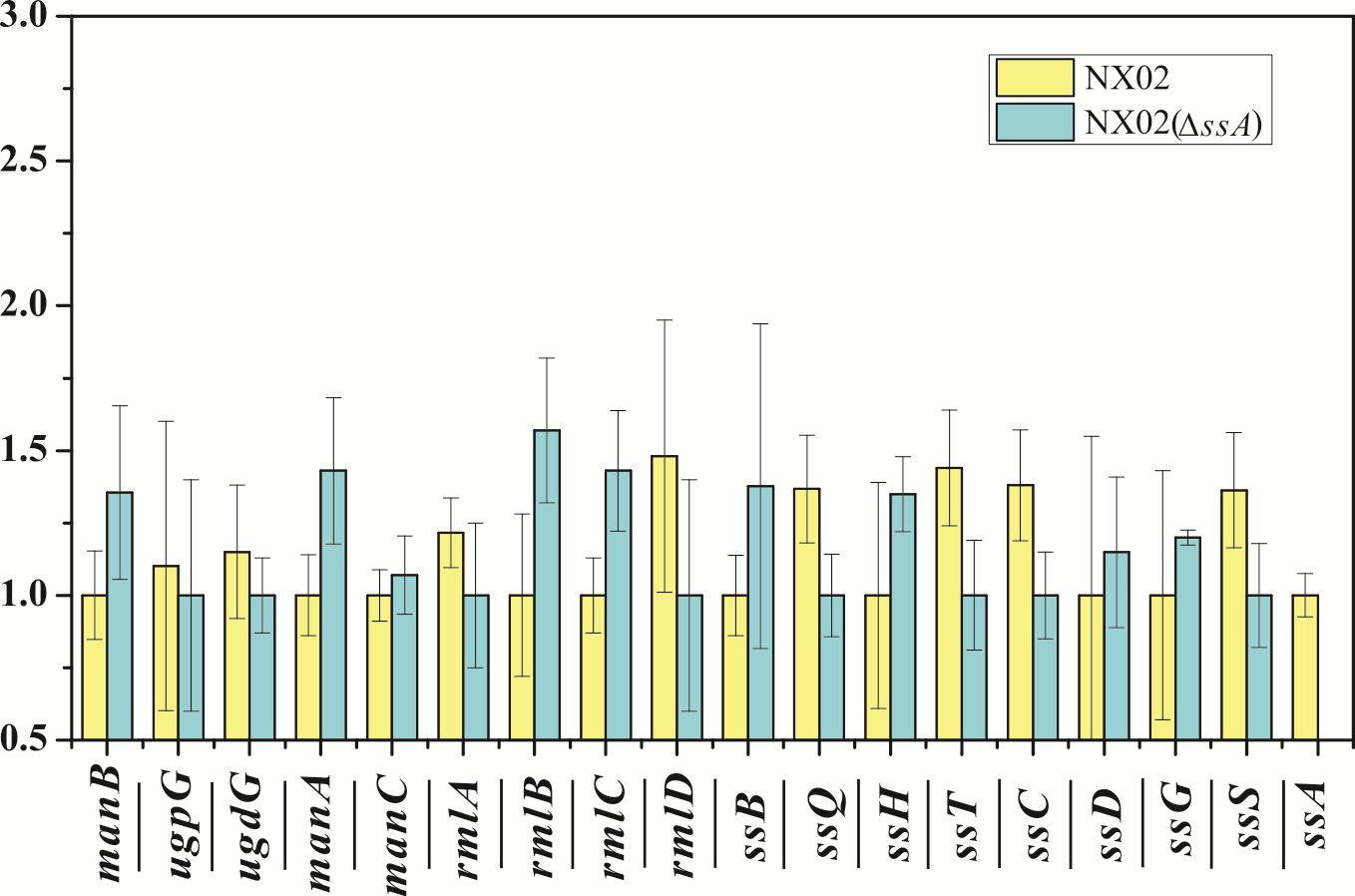
**

**Figure S4.** Relative expression levels of genes related to sanxan synthesis in wild-type NX02 and NX02 (Δ*ssA*) strains. Standard deviations were calculated from three PCR replicates and the relative abundance of genes was determined using the comparative Ct method.

**
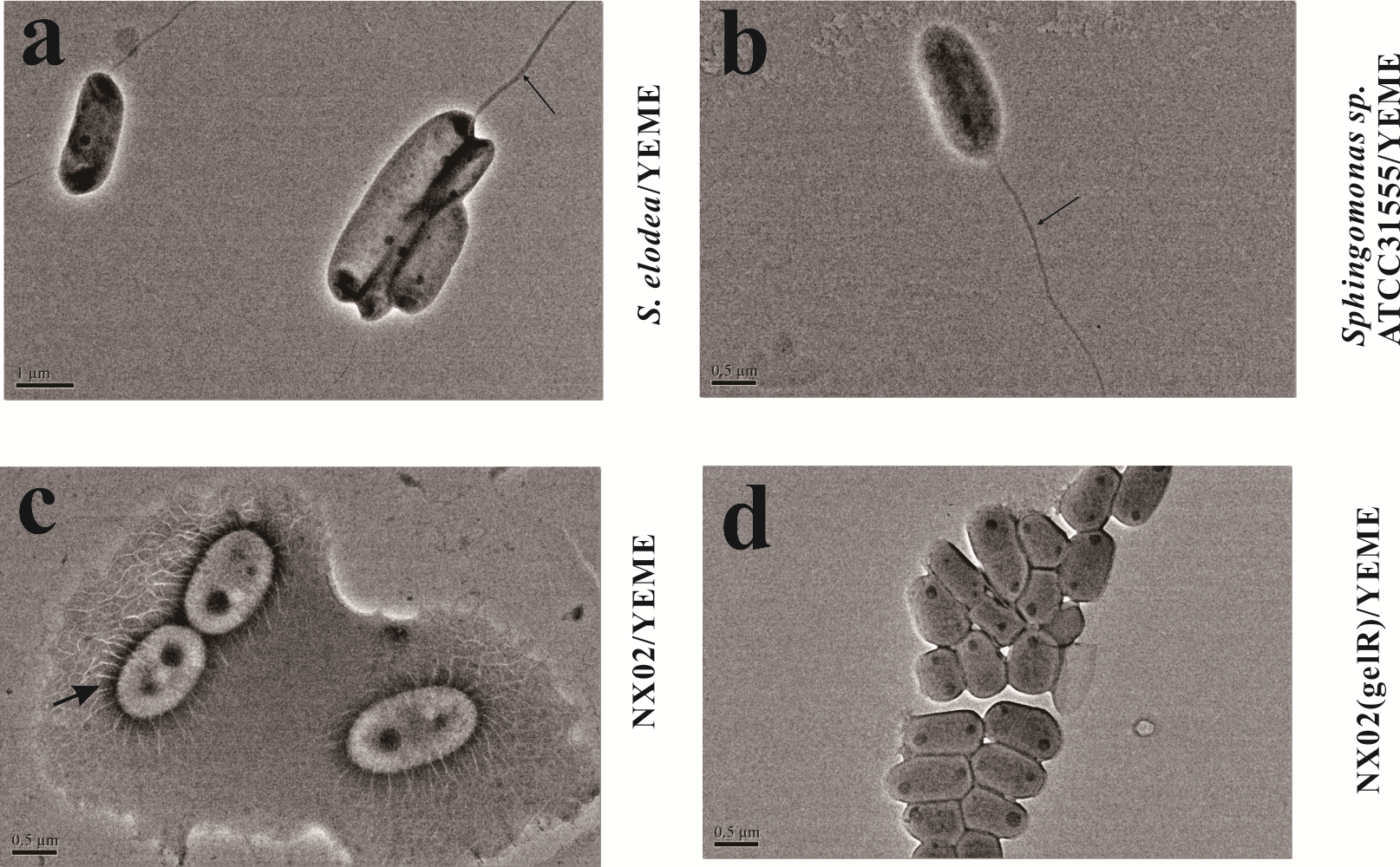
**

**Figure S5.** Ultrastructure of *Sphingomonas* strains from whole-mount samples. Polysaccharides from *Sphongomonas* *elodea* and *Sphingomonas* sp. ATCC31555 were secretory expolysaccharide (a, b), in which flagella (small arrow) were found; sanxan (bold arrow) was capsular polysaccharide that attached to the surface of outer membrane (c) and could be cut from its surface when plasmid pBBRgelR was transformed (e).

**
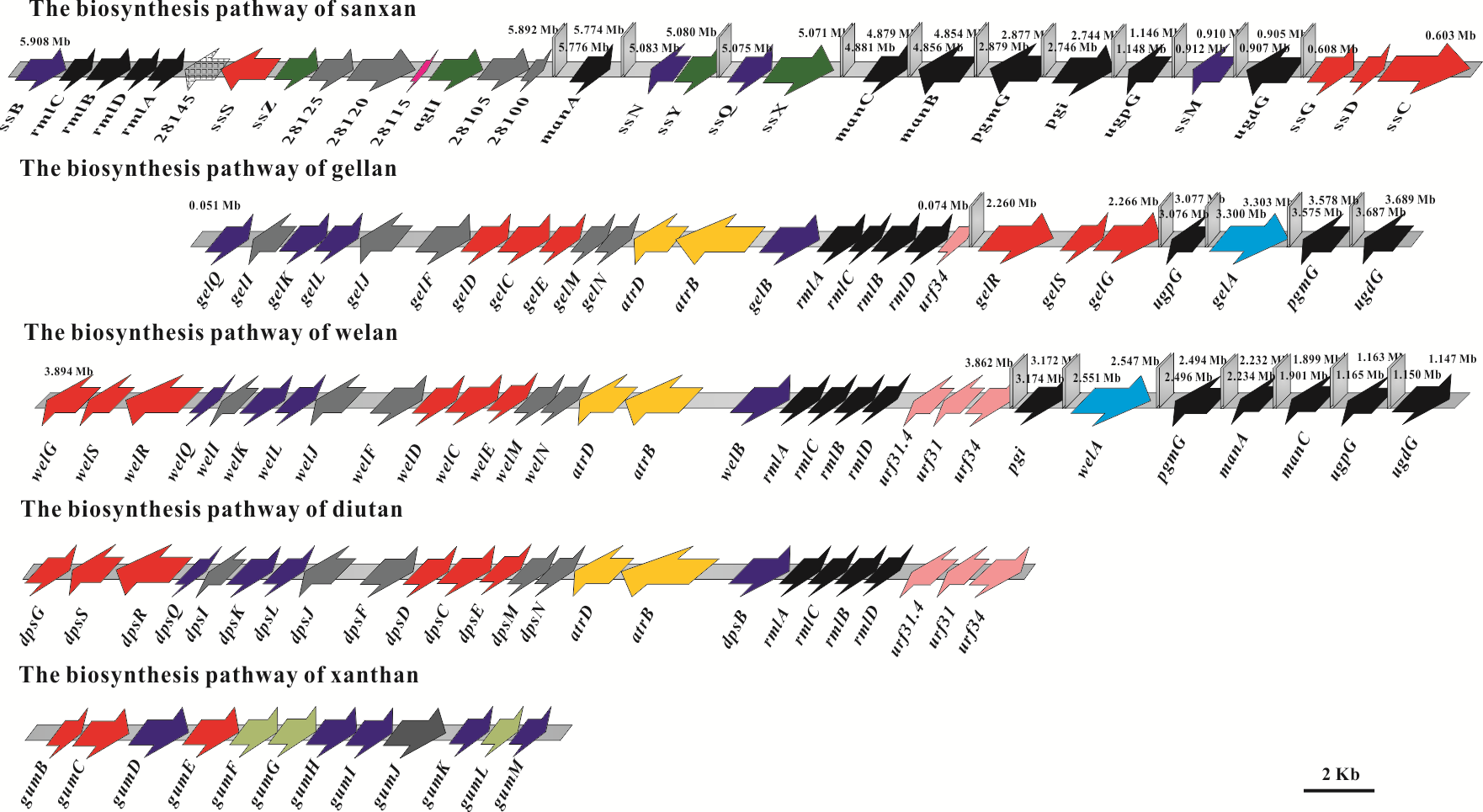
**

**Figure S6.** Comparisons of biosynthesis pathways of sanxan, sphingans (gellan, welan, diutan), and xanthan. The numbers at the top of gene clusters (sanxan, gellan, and welan) indicate the position of genes on their chromosomes
